# Computational Research on Mobile Pastoralism Using Agent-Based Modeling and Satellite Imagery

**DOI:** 10.1371/journal.pone.0151157

**Published:** 2016-03-10

**Authors:** Takuto Sakamoto

**Affiliations:** Institute of Developing Economies, Japan External Trade Organization, Chiba, Japan; University of Maryland at College Park, UNITED STATES

## Abstract

Dryland pastoralism has long attracted considerable attention from researchers in diverse fields. However, rigorous formal study is made difficult by the high level of mobility of pastoralists as well as by the sizable spatio-temporal variability of their environment. This article presents a new computational approach for studying mobile pastoralism that overcomes these issues. Combining multi-temporal satellite images and agent-based modeling allows a comprehensive examination of pastoral resource access over a realistic dryland landscape with unpredictable ecological dynamics. The article demonstrates the analytical potential of this approach through its application to mobile pastoralism in northeast Nigeria. Employing more than 100 satellite images of the area, extensive simulations are conducted under a wide array of circumstances, including different land-use constraints. The simulation results reveal complex dependencies of pastoral resource access on these circumstances along with persistent patterns of seasonal land use observed at the macro level.

## Introduction

Drylands occupy more than 40% of the earth’s land surface. These enormous areas, mostly found in Africa and Asia, are home to several hundred million pastoralists, who more or less rely on raising livestock such as camels, cattle, sheep and goats for their livelihoods [1–3]. In large areas of these drylands, rainfall patterns are highly unpredictable, rendering rangeland ecologies spatially heterogeneous and temporally variable [4–6]. In those harsh environments, the constant access to natural resources—such as pasture and water for the livestock—naturally constitutes the dominant concern for pastoralists. Anthropologists and ecologists have documented a wide variety of adaptation strategies that pastoralists have developed for attaining this difficult objective [7–12]. A high level of mobility can almost always be found in such a list of strategies. From daily herding around a temporary camping site to seasonal movements between ecological zones, pastoralists move on a varying degree of spatial and temporal scales. Despite the increasing trend toward sedentarization [13], many of them still move around extensive spaces, exploiting spatially and temporally variable resources with their livestock.

The high level of mobility over constantly changing landscapes turns pastoral societies and their surroundings into highly interactive and dynamic systems. This circumstance, in turn, has made it difficult to answer some of the essential questions about pastoral livelihoods in a rigorously formal manner: how do pastoralists move in a given spatial environment for a given period of time, and how much in natural resources do they obtain for their livestock by moving as they do? General and quantitative answers to these questions are mostly lacking in the otherwise rich and interdisciplinary literature on pastoralism [8,14,15], except for several studies that analyze pastoral resource access in limited spatial and temporal confines [16,17]. This literature gap has significant practical implications. Pastoralists around the globe have long been struggling with a variety of potentially disrupting forces, such as consolidation of the state territorial orders and expansion of agricultural lands, which can seriously constrain their land use [18, 19]. Without a solid understanding of pastoral mobility and resource access, an accurate assessment of the impact of such factors on pastoral livelihoods and their sustainability cannot be achieved.

One promising development in this regard is the recent trend toward quantitative measurement of pastoral movements and land-use patterns. Although these patterns have long been the object of detailed description among anthropologists and geographers [9,11,20,21], a growing number of researchers now utilize advanced technologies such as satellite imagery and GPS to directly measure the movements of pastoralists and their livestock [22–29]. Most of these studies focus on the movements of a relatively small number of herds at relatively small spatial and temporal scales. For example, in a recent study from northern Cameroon [26], 21 cattle tracks were obtained during dairy herding, and these samples showed a mean grazing radius of 4.4 km and a mean temporal duration of approximately 11 hours. Other studies are more extensive, in both the temporal and spatial dimensions. For example, combining direct tracking of hundreds of cattle herds with thousands of interviews with Fulani herders in western Niger, another recent study was able to reproduce some 6500 grazing itineraries across vast territories over a two-year period [30]. In principle, these methods can be employed in other cases for the rigorous examination of pastoral mobility on different spatio-temporal scales.

However, these largely data-driven approaches, which are based on direct measurement of pastoral movements, have their own limitations. From characteristic climate patterns to dominant socio-economic institutions, dryland pastoralists live in quite diverse contexts. Even in the same place, substantial inter-annual and intra-annual variations in the rainfall, which drive the ‘non-equilibrium’ ecological dynamics [4–6,31], constantly force adjustments and adaptation on their behavior. These factors show that an enormous amount of tracking data, which is extensive both in temporal and spatial dimensions, might be necessary to derive any robust statement about pastoral mobility and resource access. Given the sheer amount of data that is required, the limited number of grazing itineraries obtained from limited temporal and spatial confines would be likely to be incomplete, whereas efforts to keep up with those requirements would be equally likely to be costly and, perhaps, prohibitively costly.

Thus, a complementary and, at the same time, more cost-effective approach is needed along with the data-driven methods discussed above. This article presents such an approach. The approach builds on the combined use of two computational methods: agent-based modeling and satellite image analysis. The former makes it possible to explicitly simulate pastoral movement patterns under different circumstances and to compute the resultant resource access that pastoralists can achieve under each circumstance. The latter generates a series of empirical spatial data that adds realism to the simulations mentioned above, with monthly spatial distributions of dryland vegetation over an extended period of time. Thus, the present approach embraces a higher level of spatial and temporal variability in rangeland ecologies than previous approaches allow, and, on this basis, enables one to rigorously examine pastoral movements and resource access in a wide range of possible situations. The article concretely demonstrates these advantages by applying the methods to the analysis of mobile pastoralism in northeast Nigeria.

Computational modeling, including the agent-based modeling employed here, has always had some limited presence in the literature on pastoralism [32–37]. In this methodological tradition, the present study has several distinctive characteristics. First, it has a sharp focus on pastoral mobility and land use, which necessarily entails a spatially explicit modeling approach. Second, among the models that have similar substantive focus and methodological orientation [38–42], the model presented here achieves a fuller level of integration with empirical spatial data, especially those derived from satellite imagery. This makes it possible to tap the fruits of a growing body of research that attempts to derive the patterns and dynamics of dryland ecologies from different sources of satellite imagery [43–48]. Third, while pursuing the empirical relevance in this way, the model still retains a simple structure and behavioral logic like some of the formal models that deal with spatial foraging behavior in general [49,50]. This greatly helps to achieve a clear theoretical understanding of the process that governs pastoral movements and resource access.

This article is organized as follows. The next section details the suggested approach, describing the agent-based model of mobile pastoralists and the satellite-derived datasets used as inputs to the model. Taking northeast Nigeria as a focus area, the section then introduces multi-temporal data on the vegetation distribution in the area and lays out a plan for simulations that employ these data. The ensuing section reports the main results of these simulations. Among them is the highly structured spatio-temporal pattern of land use that the model generates in a wide range of parameter conditions. The section describes this pattern along with other aspects of the model’s behavior and derives their implications for understanding mobile pastoralism in northeast Nigeria. Some of the major issues that are threatening the pastoralism there, including massive cropland expansion, are also examined in this context. Last, the final section concludes the arguments and suggests a further step to consolidate the approach.

## Materials and Methods

The basic idea behind the present approach is as follows: First, we construct simple mobile agents that exhibit, subject to stochastic perturbations, reasonable adaptive responses to the surrounding environment and its changes; second, we allow these virtual agents to move around, interact with each other, and adapt themselves in the spatial environment that is modeled based on the realistic ecological dynamics that are derived from a series of satellite images; and third, we replicate this process many times and in a wide array of parametric settings to obtain variation with regard to the movement patterns and resource access of the agents. The result is a firm basis for the understanding of mobile pastoralism, which can be further consolidated with the complementary use of other existing approaches, including detailed tracking of actual pastoralists (see the last section).

### Model Description

A simple agent-based model describes the adaptive behavior of mobile pastoralists and formalizes their interactions among themselves as well as with a dryland environment. The virtual agents that represent pastoralists are hereafter called NOMADs, while the simulated environment is called ENV. This model is coded in Python 2.7.5 and is downloadable from GitHub [51]. The model requires some of the widely used Python extensions for its running: SciPy (0.15.1), Matplotlib (1.4.3), and the GDAL Python package (2.0.0). Its implementation partially depends on a Python-coded simulator that was developed in [52], which is also publicly available online [53].

**Fig 1:**
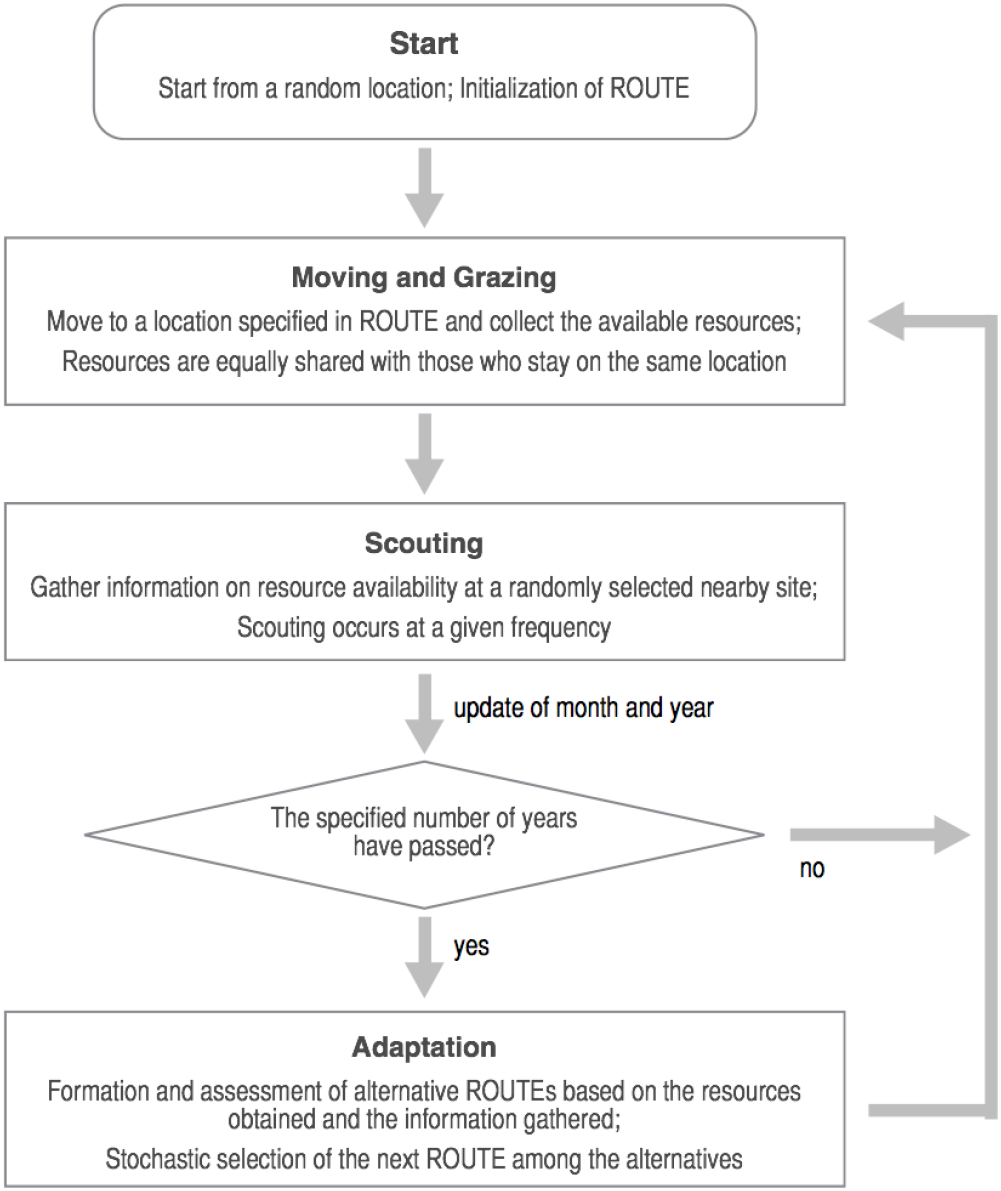
The flow of the NOMAD behavior.

In the model, ENV is a two-dimensional grid space that represents a certain tract of dryland. It has a configuration of grazing resources that is both spatially and temporally variable. The distribution and dynamics of these resources are derived from empirical data in the way described in the next sub-section. NOMAD agents move around in this potentially variable, uncertain environment, seeking grazing resources for their livestock, whose existence is implicitly assumed in the model. Actual pastoralists, especially their highly mobile portion, constantly move over an extensive area of rangeland in response to seasonal and yearly changes in the availability of pasture and water around their locations [4,9–11,20,21]. To ensure that their livestock gets the best grazing locations available, these pastoralists closely monitor rangeland conditions in the surrounding landscape and sometimes even dispatch ‘scouts’ to seek information that is further afield [9,11]. Based on the pieces of information that are thereby collected and stored, the pastoralists adaptively change their movement patterns over time. The behavioral rule of a NOMAD, whose flow is illustrated in Fig 1, concisely captures these most essential aspects of mobile pastoralism.

The key component of this rule is a ROUTE, which is a planned route of a NOMAD’s monthly movements over a year. Each movement of each agent takes place while the agent follows its ROUTE month by month, whereas the adaptation of the agent to changing conditions in ENV occurs through an update of this ROUTE at some intervals of years. Reflecting the generally closed nature of transhumance paths followed by actual pastoralists, a ROUTE is formulated as a single cycle (denoted as ρ) that connects a series of two-dimensional integer coordinates (12 in total): each coordinate denotes the location of a grid cell where the NOMAD concerned stays each month of the year. A simulation run starts from a somewhat extreme situation in which each agent is ‘settled’ in a random location in ENV. For example, if an initially assigned location for a NOMAD is (6, 5), then the initial ROUTE for this agent becomes a repetition of this set of numbers, (6, 5)→(6, 5)→…→(6, 5), from January to December.

Two sources of information strongly condition the ROUTE updating process: grazing and scouting. First, a monthly visit to a specific location and the ensuing grazing activities there give a NOMAD firsthand information about how much resource is available around the area at a particular time. Specifically, each agent assesses the condition of a visited location during the month of the visit by computing a mean value of the resources available within some specified range (GRAZE RANGE) around that location in that month. Second, with a specified probability per month (SCOUTING FREQUENCY), a NOMAD also scouts the surrounding landscape to collect the information on local resource availability. Then, the NOMAD randomly selects one site within a specified distance (SCOUTING RANGE) from its grazing site and assesses the condition of the selected site and its surrounding area largely in the same way as in the case of grazing.

In either case, if the agent has visited or scouted the same site in the same month before, the gathered information is integrated with that of the preceding visit, and the recorded resource availability becomes the time-mean value. Moreover, there can be an overlap of grazing areas between two or more NOMADs. In this case, the calculation of resource access for each of the agents concerned is adjusted in such a way that the resources in the overlapped area are equally shared among them. For example, if three agents graze one site with a resource value of 0.6 in the same month, then the amount of resources that are available to each of the agents in that month is computed to be 0.2. On the other hand, too many agents can degrade ENV itself. Each site in ENV can host a certain number of NOMADs over a year (CARRYING CAPACITY). If the accumulated number of NOMADs that have visited a site exceeds this capacity, then the available resources there become zero in the remaining months of the year concerned. These simple rules exhaust the possible direct interactions among NOMADs and ENV.

Next, the ROUTE update process proceeds as follows. At specified intervals of years (INTERVALS OF ROUTE UPDATE), a NOMAD selects its next ROUTE among the specified number of alternative yearly paths (NUMBER OF ALTERNATIVE ROUTES). The alternatives include the existing ROUTE that the agent has followed thus far; otherwise, they are generated through a random combination of the sites that the agent already visited or scouted. Each of these alternatives is then assessed through the calculation of its potential. Formally, the potential *H*(ρ_i_) for the i-th alternative path ρ_i_ is a linear combination of the (negative) expected availability of grazing resources and the costs incurred during the agent’s movement along the path, as the following equation represents:

$$H\left(\rho_{i}\right)=\alpha \left(-\sum_{m=1}^{12}R_{m}\left(\rho_{i}\left(m\right)\right)+\sum_{m=1}^{12}C\left(\rho_{i}\left(m\right),\rho_{i}\left(m+1\right)\right)\right)+\beta$$ (1)

In Eq 1, ρ_i_(m) denotes a site that ρ_i_ dictates the agent to visit in the m-th month, assuming ρ_i_(13) = ρ_i_(1). The function *R*_*m*_ returns the time-mean of the grazing resources that are available around that site in the m-th month, which is directly derived from the information that the agent has gathered in the way mentioned above. The function *C*, on the other hand, computes the movement cost that is incurred while the agent moves between two adjacent sites along ρ_i_. *C* is formulated here in the simplest possible way: if the distance between the two adjacent sites exceeds a certain mobility threshold (MOVE RANGE), then the agent suffers a very large ‘penalty’ (PENALTY TO MOVEMENT BEYOND RANGE). The overall movement cost along ρ_i_ is the sum of these penalties, which effectively discourages physically unrealistic ROUTEs. Last, α and β are scaling parameters. In the following simulations, they are fixed at 2.0 and 1.0, respectively.

The selection of a new ROUTE takes place stochastically using the following probability distribution *P*(ρ_i_):

$$P\left(\rho_{i}\right)=\frac{\text{exp}\left(-H\left(\rho_{i}\right)/E\left(H\left(\rho_{1}\right)\right)\right)}{\sum_{j=1}^{N}\text{exp}\left(-H\left(\rho_{j}\right)/E\left(H\left(\rho_{1}\right)\right)\right)}$$ (2)

Function (2) gives the probability that among all of the alternatives considered (*N* in total), the agent will adopt a path ρ_i_ as its next ROUTE. *E* controls the amount of random perturbation (STOCHASTIC NOISE) that is applied to this ROUTE selection. It depends on the potential of the current ROUTE (denoted as ρ_1_), which allows dynamic and flexible adaptation. Here, a crude form of an ‘annealing’ process is assumed for the adaptation of each agent: the noise *E* takes a minimum value (1.0^-6^ in the following simulations) whenever the potential *H*(ρ_1_) becomes sufficiently small (0.0), while *E* takes a maximum value (1.0^-2^) whenever *H*(ρ_1_) reaches a certain high value (1.0). The dependence is linear between these two extremes.

The NOMAD rule described so far represents the micro-behavior of actual pastoralists in a highly abstract fashion. The rich ethnological literature on pastoralism reveals the latter’s substantial diversity and complexity [9,11,12,14,15,20,21,54]. Thus, various alternative specifications of the model are naturally conceivable. For example, there are few empirical reasons to believe that actual pastoralists compute costs incurred during their movements in the way that the assumed functional form of *C* implies; other forms such as linear dependence on the distance moved are at least equally plausible. Similarly, the assumption that the agents automatically share grazing resources whenever they stay on the same site might sound somewhat unrealistic given the prevalence of intense resource competitions among pastoral communities in some places including East Africa [55,56,57]. Therefore, the model’s rule needs to be constantly adjusted against the specific context to which it is applied. It would also be helpful to actually implement and test alternative specifications of the model to examine the effects of their differences on the model’s macro-behavior. Some preliminary results of such an investigation are reported in Supporting Information (S1 Table).

### Input Data Description

Empirical distributions of grazing resources can be measured by different vegetation indices, which are computed from multi-spectral reflectance measurements that are derived from remote-sensing data. Among these indices, the following Normalized Difference Vegetation Index (NDVI) is the most widely used [48, 58]:

$$NDVI=\frac{NIR-R}{NIR+R},$$

where *NIR* and *R* stand for the spectral reflectance in the near-infrared and visible red bands, respectively. NDVI varies between -1.0 and +1.0 and measures the ‘greenness’ of the vegetation that is found in a given pixel of a remotely sensed image. This surprisingly simple formula has been shown to have strong correlations with various quantitative aspects of vegetation resources on the ground, including photosynthetic capacity, herbaceous biomass and vegetation cover, which have extensive applications to dryland ecologies [23,44,45,59–63]. As vegetation growth in these ecologies is known to closely follow rainfall amount [6,64], NDVI can also be employed as a useful surrogate for the availability of water resources, especially at relatively large spatial and temporal scales [65].

In the simulations below, the NDVI dataset derived from MODIS (Moderate Resolution Imaging Spectroradiometer) satellite imagery is employed to form resource distributions in ENV. MODIS, a multispectral sensor aboard the Terra and Aqua satellites, captures each area on the earth at a relatively high frequency (a maximum of four times per day), making it an ideal data source for temporally fine-grained simulations such as those conducted here. NASA (National Aeronautics and Space Administration) provides a wide range of spatial datasets that are processed from MODIS imagery, including vegetation index products [66]. These datasets are publicly available at USGS (United States Geological Survey)’s EarthExplorer portal [67]. The specific dataset used is Vegetation Indices Monthly L3 Global 1 km (MYD13A3) [68]. This dataset contains a series of composite NDVI data with a spatial resolution of 1 km x 1 km per pixel and a temporal resolution of one month. The monthly composite data were selected over temporally more resolute data products because the former effectively removes the distorting effects of clouds that the original images might contain. Furthermore, the map projection is sinusoidal. These data properties remain unchanged in the following simulations, whereas the original format of the MODIS products, HDF-EOS, is converted to the GeoTIFF format using multispectral image analysis freeware called MultiSpec 3.5 [69]. The same software is employed for processing the GeoTIFF outputs that are derived from the simulation runs.

Although MYD13A3 is a highly processed, generally reliable dataset on the vegetation conditions on earth, some adjustments are needed for its use as an input to the agent-based model of mobile pastoralism. NDVI mostly measures quantitative aspects of vegetation resources such as biomass. Pastoralists and their livestock, on the other hand, have specific preferences for these resources, which cannot easily be captured by the quantitative aspects alone. For example, cattle herders most of the time prefer grasslands to woodlands, even though the latter generally show higher NDVI values than the former. To address this consideration and other types of discrepancies, two additional steps of processing are applied to the NDVI data.

First, negative values of NDVI are set to 0.0. These values typically indicate residual elements of cloud cover. Otherwise, given the spatial resolution of the data, they denote relatively large areas of water or snow surface, which are either inaccessible or irrelevant to mobile pastoralists. In any case, as far as the present study is concerned, the corresponding sites remain a quite tiny portion of the total pixels (0.1–0.2%). Second, NDVI values are allowed to be adjusted depending on the land cover type of the site that is concerned. More specifically, NDVI values at the sites whose land types are not preferred by pastoralists are discounted by some specified factor. Here, another MODIS data product is utilized: Land Cover Type Yearly L3 Global 500 m SIN Grid (MCD12Q1), which classifies each pixel (at a resolution of 500 m) over a one-year composite of MODIS images according to major land classification criteria [70]. Among the five different classification schemes that are covered in this dataset, the University of Maryland (UMD) scheme (Land Cover Type 2) provides a sufficiently detailed and relevant land cover classification. In the simulations described below, the NDVI values are uniformly reduced by 50% unless the sites concerned are classified as ‘grasslands’, ‘savannas’, or ‘woody savannas’ in the UMD scheme (see S1 Table for other specifications). The procedure for these adjustments is fully integrated with the simulation model and is provided with various control options.

### Simulation Examples

**Fig 2:**
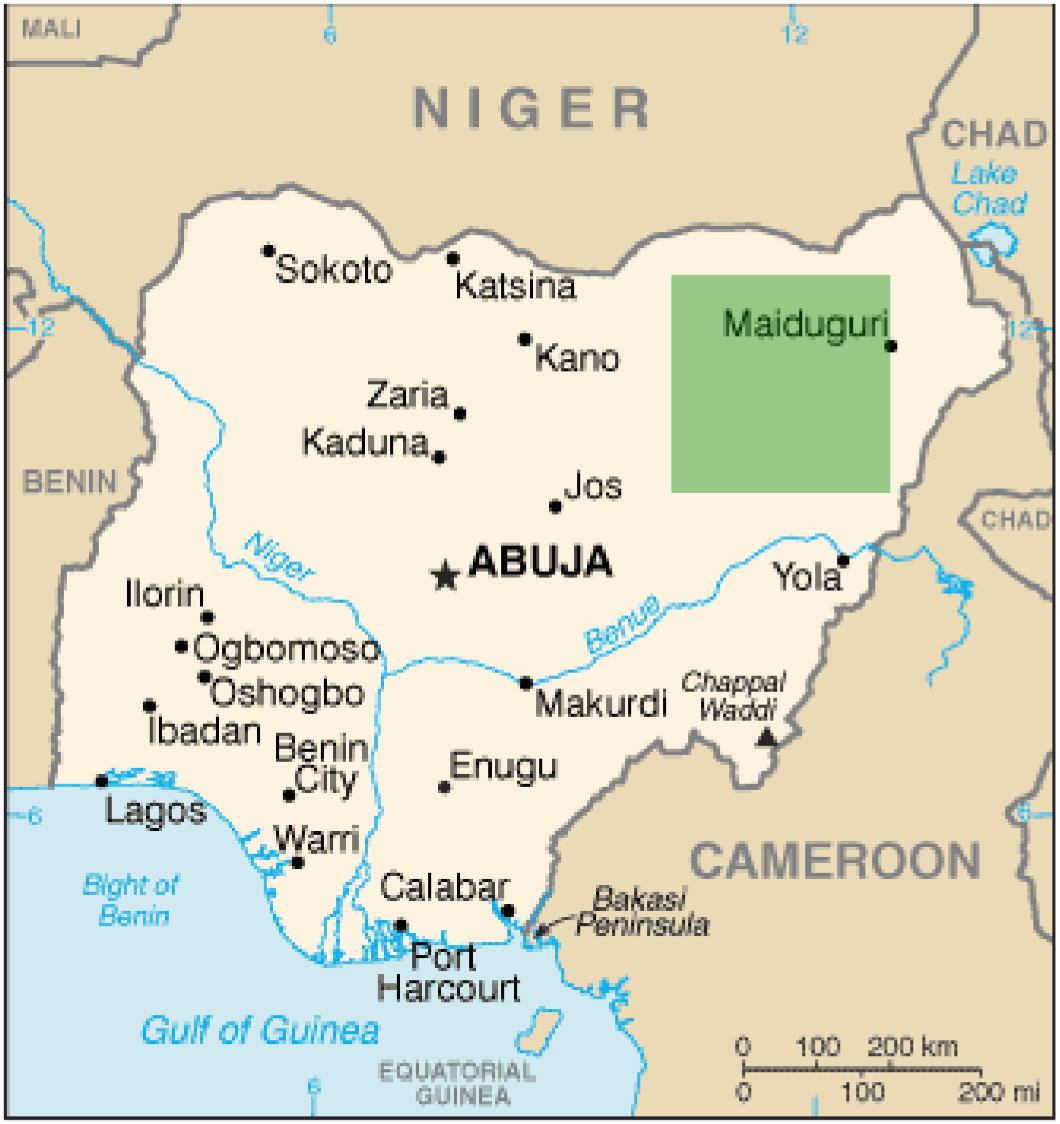
The area of study. The shaded square indicates the study area. The reference map of Nigeria was taken from the CIA’s World Factbook (https://www.cia.gov/library/publications/the-world-factbook/geos/ni.html).

Given the near-global coverage of the MODIS data products, the simulation model can be run in almost any geographical setting. In the following, the area focus is on a part of northeastern Nigeria: the 300 km x 300 km area that is indicated in the map in Fig 2. This mostly semiarid area, which spans the Sudano-Sahelian and Sudanian ecological zones from the north to the south, has long been home to Fulani pastoralists. In fact, it contains one of the study areas in which British anthropologist Stenning conducted his classical work on Fulani transhumance more than 60 years ago [21,71]. Other researchers also wrote extensively about Fulani pastoralists in different places at different times [20,22,72–74]. These studies can be brought together to evaluate the general plausibility of the model’s behavior.

**Fig 3:**
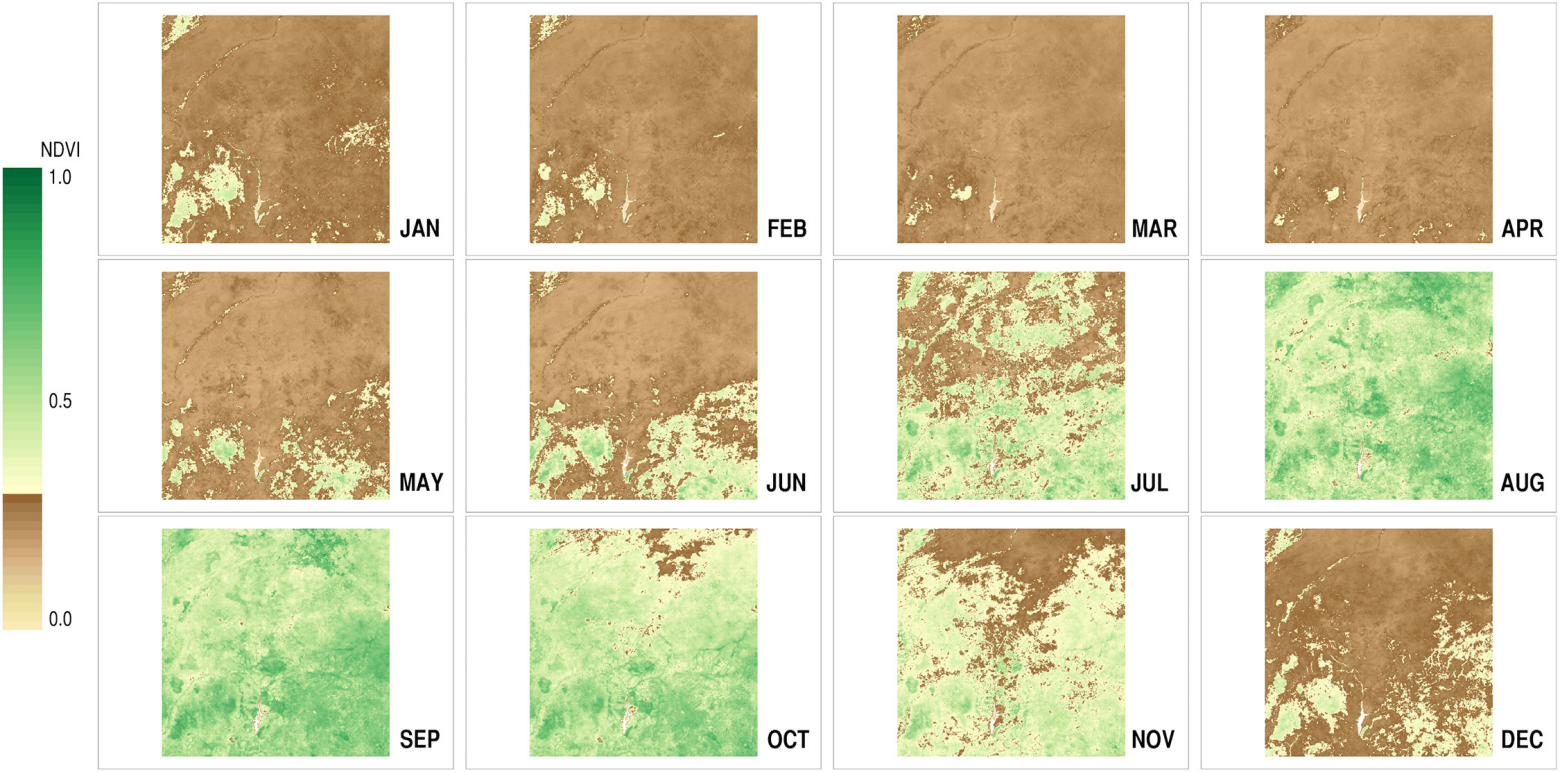
Monthly vegetation changes in 2014. These maps were generated from the MODIS NDVI dataset (MYD13A3). The greener pixels indicate the sites that have more abundant vegetation, while the browner pixels correspond to those that have sparser vegetation.

Many of the MODIS data products are available for the period from 2000 to the present. The NDVI data (MYD13A3) that covered all of the months from January 2005 to December 2014 (120 months in total) were obtained for the area concerned (panel ID: h19v07). Fig 3 displays the successive monthly distributions of the NDVI values in the area in the year 2014. The temporal coverage of 10 years, which is the typical cycle of severe (often successive) droughts in the Sahel [45], actually includes the two years that saw major regional droughts (2010 and 2012). This coverage with the resolution of one month ensures that sizable inter-annual and intra-annual variations are incorporated into ENV’s ecological dynamics. These data on vegetation conditions were then processed and adjusted in the way that was explained in the previous sub-section. The land cover classification data (MCD12Q1) for the year 2010, the mid-point of the 10-year period, was referenced in this process. The resulting series of 120 monthly data wholly describes the spatial patterns and the temporal dynamics of the resource distribution in ENV (see S1 Table for other specifications). In simulation, this data stream is repeatedly fed into the model, returning to the first data (January 2005) after reaching the last data (December 2014). These input data are packaged with the model implementation files.

In the realistic, highly variable environment thus created, the model was repeatedly run while extensively changing the relevant parameters. The main outputs here are the movement patterns of the NOMADs and the resultant resource access for these agents. With respect to the movement patterns, particular attention was paid to the spatio-temporal distributions of land-use intensity, which emerged from long-time adaptation of the agents through their ROUTE updates. The latter was directly measured by the mean amount of grazing resources that the NOMADs obtained by following their ROUTEs. In each combination of parameters, these outputs were sampled and then aggregated over 20 runs, each of which consisted of 20,000 years (240,000 months) of interactions seeded with a different series of pseudo-random numbers. The choice of 20,000 years, or 2,000 iterations of the 10-year cycle of vegetation dynamics, ensures the NOMADs sufficient opportunities to adapt themselves to the 10-year stream of NDVI distributions. This in turn helps one to obtain reliable estimates of the model’s underlying dynamics in a diverse array of settings; it does not imply an unrealistic assumption that the agents, much less actual pastoralists, need exactly this amount of time to evolve their characteristic behavioral traits. Actual speed of adaptation can vary considerably depending on the parameter values. For example, in the baseline simulations detailed below, the main outputs of the model such as the mean amount of resources typically began to show some regularity within the first thousand years.

Table 1 lists the main parameters of the model along with their values used in the simulations. The underlined numbers denote a combination of the baseline values, for which the model’s behavior was closely observed and analyzed. Many of the parameters were also extensively manipulated to examine their effects on the output variables. More specifically, each of these parameters was changed to one of the alternative values listed in the second column of the table while the other parameters were held constant at their baseline values. This piecemeal check of the effects of the parameters, while being far from exhaustive as a sensitivity analysis, nevertheless allows a careful examination of causal mechanisms involved in the behavior of the agents and their interactions. Moreover, as is shown below, such an analysis can still establish some broad patterns regarding the model’s macro-behavior that are observed over a fairly wide range of possible circumstances. These patterns, if consistent with the available empirical observations, will offer solid arguments about pastoral mobility and resource access in the area concerned.

**Table 1:** The main parameters of the model.

Parameters	Values^a^
**NOMAD POPULATION**	{1, 2, 5, 10, 20, 50}
**MOVE RANGE (km per month)**	{30, 50, 80, 100, 130, 150}
**GRAZE RANGE (km)**	{0, 1, 2, 3, 4, 5, 10}
**SCOUTING FREQUENCY (per month)**	0.2
**SCOUTING RANGE (km)**	100
**CARRYING CAPACITY (agents per year)**	{1, 4, 7, 10, 13}
**INTERVALS OF ROUTE UPDATE (years)**	1
**NUMBER OF ALTERNATIVE ROUTES**	{2, 10, 50, 100, 200}
**PENALTY TO MOVEMENT BEYOND MOVE RANGE**	100
**STOCHASTIC NOISE (Minimum)**	10^-6^
**STOCHASTIC NOISE (Maximum)**	{0.001, 0.002, 0.005, 0.01, 0.02, 0.05, 1.0}

^a^ Each of the listed values was tested in the simulations reported below. The underlined numbers are baseline values.

In addition to changing the basic parameters of the model, the study also examined the effects of two large model extensions: the disruption caused by tsetse flies, which are major livestock disease vectors in tropical Africa, and the land-use pressure coming from agricultural land expansion. These are highly visible constraints on pastoral mobility in contemporary West Africa [73–76]. The model allows one to rigorously assess their effects on pastoral livelihoods as well as draw some practical implications for enhancing the sustainability of pastoralism.

**Fig 4:**
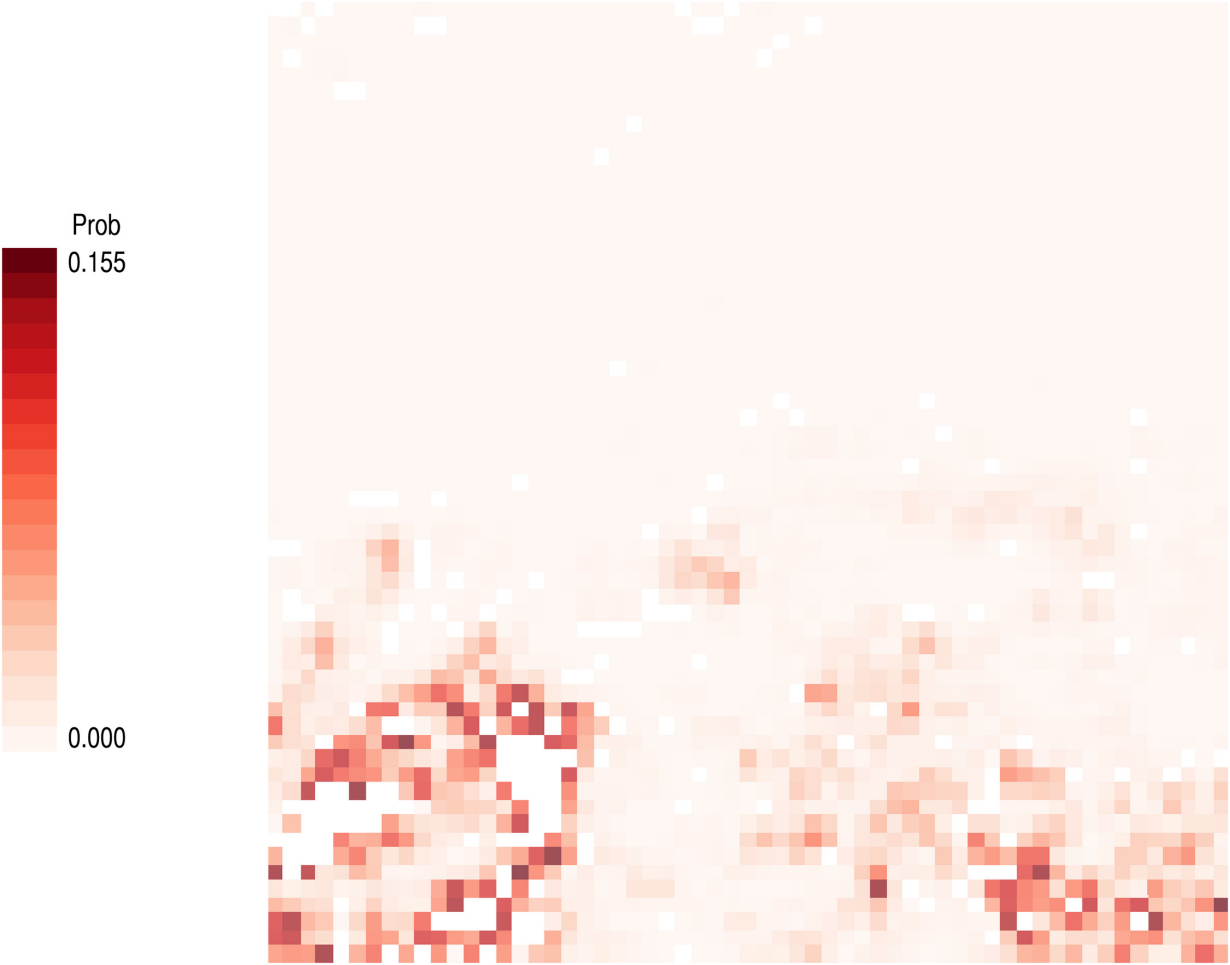
Predicted distribution of tsetse flies (*Morsitans* group). This map was generated from FAO’s GIS dataset on tsetse fly distributions. Darker red pixels correspond to the areas that have a higher probability of tsetse presence.

Regarding tsetse flies, the Food and Agricultural Organization (FAO)’s Programme Against African Trypanosomosis (PAAT) provides a publicly available geo-spatial dataset on their distribution in Africa [77]. This dataset, which incorporates diverse sources of information gathered in different countries in different periods, essentially consists of probability maps showing the presence of various species of tsetse flies at a somewhat crude spatial resolution of 5 km x 5 km [78]. Here, the predicted distribution of the so-called ‘savanna flies’ (Subgenus *Morsitans*), which includes five different tsetse species such as *Glossina austeni* and *Glossina morsitans*, was employed as an additional input to the simulation. Using a freeware QGIS 2.8 [79], the projection and resolution of the original data were made to be compatible with the MODIS datasets. Specifically, in the rescaling of the spatial resolution from 5 km x 5 km to 1 km x 1 km, the predicted probability of tsetse presence on each site in the original data was up-sampled so that the per-area probability remained the same between the site and each of its corresponding rescaled sites. The resulting map, which is depicted in Fig 4, assigns to each site in ENV the probability that a NOMAD encounters tsetse flies whenever the agent grazes or scouts the site. The experienced frequency of these encounters now constitutes another component of the information that is of concern on the site, along with resource availability. The weight of this added component in an agent’s assessment of ROUTEs is represented by DISRUPTION EFFECT, which is another parameter that appears in the expanded version of the potential function *H*(ρ).

**Fig 5:**
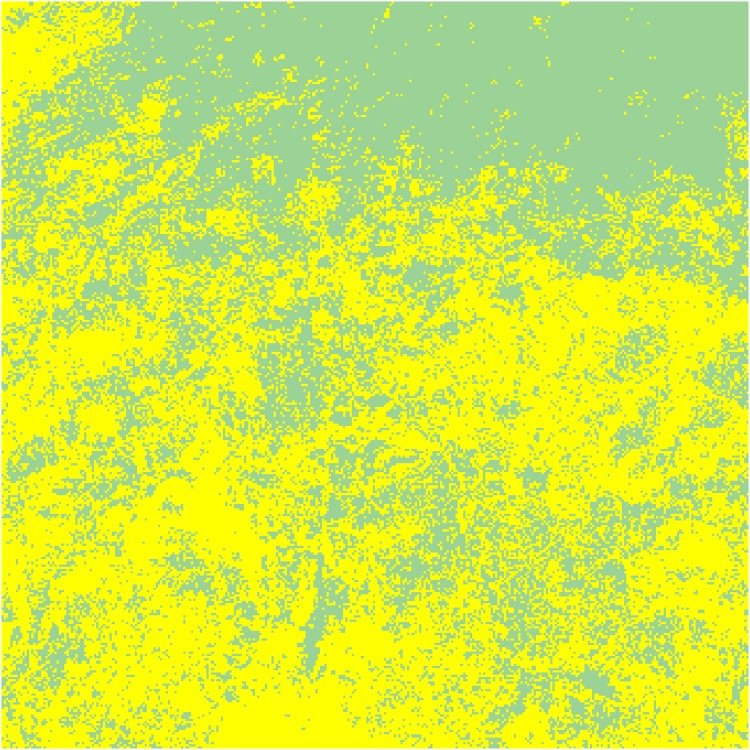
Distribution of croplands in 2010. This map was generated from the MODIS land classification dataset (MCD12Q1). The yellow pixels correspond to the sites that are classified as ‘croplands’ according to the UMD classification scheme. The green pixels indicate the other land classes, such as ‘grasslands’ and ‘savannas’.

Last, land-use constraints can be imposed on NOMADs by denying them access to vegetation resources on a specific type of site in ENV. In the simulations reported below, NOMADs were deprived of access to ‘croplands’ in the UMD classification scheme, again using the MODIS land classification data (MCD12Q1) for the year 2010. Among the 104,976 sites that constitute ENV, the corresponding sites occupy more than 53% (55,728 sites): a vast expanse, as Fig 5 confirms. Moreover, the denial of resources can be made to be time dependent, which enables temporal control of land use. For example, as is occasionally observed in West Africa [14,74], NOMADs can be allowed into ‘fallow’ lands during several months in the dry season while being denied access to the same lands during the other months.

## Results and Discussion

### Baseline Runs

**Fig 6:**
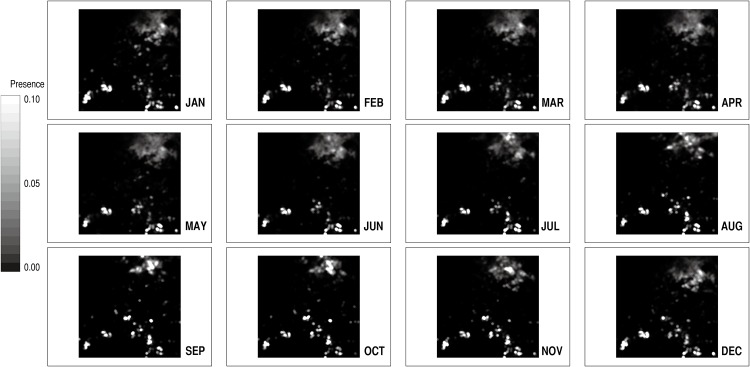
Monthly distribution of land-use intensity (baseline). These 12 maps were derived from 20 baseline runs. The parameters were given the values that are underlined in Table 1. In each of the maps, the whiter pixels indicate the sites that have a larger presence of NOMADs in the corresponding month. In this example, the maximum number of agents per pixel per year is approximately 0.798, which was recorded in June.

Fig 6 illustrates the typical behavior of the model, which was derived from the baseline runs that were conducted under the combination of parameter values underlined in Table 1. The figure depicts monthly changes in the spatial distribution of land-use intensity as measured by the average number of NOMADs per year that grazed a given site in a given month during the 20,000 years of interactions. Each of the 12 maps is a composite map that averages the results that were obtained from 20 runs. This aggregation is justified because the land-use patterns that were observed in individual runs are mostly consistent with one another. In these maps, it is fairly easy to recognize the broad configuration of land-use intensity that is characterized by two separate, contrasting geographical clusters: the smaller, somewhat diffusive cluster in the northeast (C1) and the more distinct, more extensive cluster in the south (C2).

**Fig 7:**
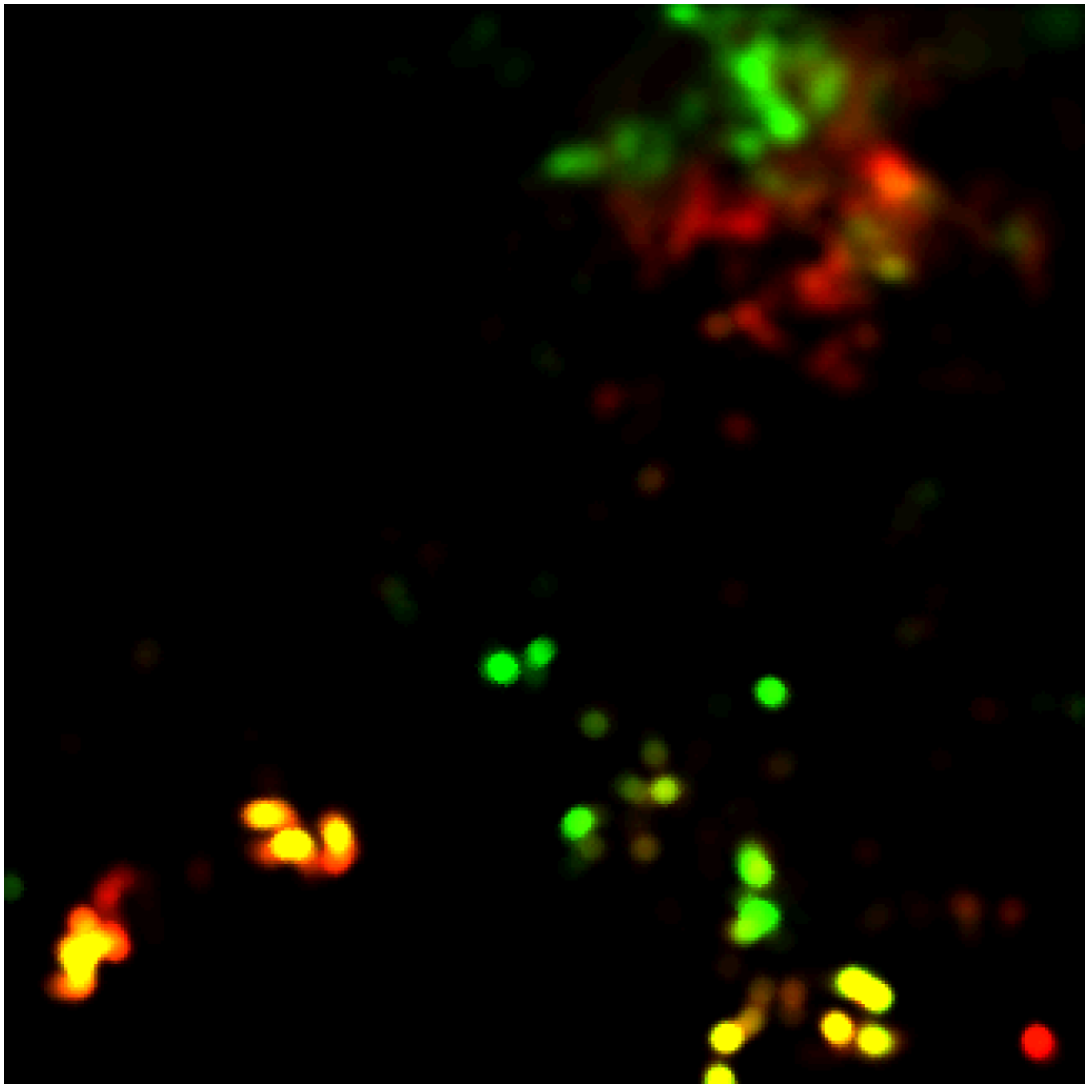
Seasonal differences in land-use intensity (baseline). This map is a composite image of the dry season (January, February and March) and rainy season (July, August and September) distributions of land-use intensity. The red pixels indicate the dominance of the dry season land use. The green pixels, in contrast, indicate the rainy season dominance. The yellow pixels denote the land use in which there is no clear seasonal difference in the intensity.

Fig 6 also indicates the sizable seasonality that is contained in the land-use pattern of the NOMADs. Fig 7 concisely captures this seasonality. This figure was derived from the preceding figure by the following two-step procedure: computing the dry season (which consists here of January, February and March) and rainy season (July, August and September) distributions of land-use intensity by averaging the corresponding monthly distributions and, then, generating a composite image of the two seasonal distributions by assigning the red band and green band in the RGB channels to the dry season and rainy season parts, respectively. Thus, the red sites indicate the area that was predominantly visited and grazed in the dry season, while the green sites indicate the area that was predominantly exploited in the rainy season. The yellow area, on the other hand, saw almost equal intensity of seasonal land use, while the black area was mostly unexploited by the agents in the first place.

This visual representation can be enhanced with a quantitative scheme for more explicit land-use categorization. One such scheme takes the following form: If the mean frequency of NOMAD visits to a site in the dry (wet) season exceeds that in the wet (dry) season by more than some threshold amount, then the concerned site will be categorized as ‘dry (wet)-season dominant’; otherwise, if the mean frequency of visit exceeds another threshold irrespective of seasons, then the site will be categorized as ‘not seasonal’; otherwise, the site will be classified as ‘not exploited’. The second threshold is held constant at 0.001. Then, several specifications (0.001, 0.01 and 0.1) of the first threshold, which is hereafter called the ‘land-use threshold’, generate different versions of seasonal land-use classification as summarized in Table 2. This information will later be used to quantitatively assess the similarity of land-use patterns among different sets of simulation runs.

**Table 2:** Seasonal land-use classification in the baseline condition.

Threshold	Dry Season	Wet Season	Not Seasonal	Not Exploited	Total
0.001	15819 (15.07%)	7992 (7.61%)	3927 (3.74%)	77238 (73.58%)	104976
0.01	5263 (5.01%)	4191 (3.99%)	18284 (17.42%)	77238 (73.58%)	104976
0.1	157 (0.15%)	406 (0.39%)	27175 (25.89%)	77238 (73.58%)	104976

Turning back to Fig 7, C1 in the northeast shows a rather clear seasonal north-south displacement. Rainy season land use extends toward the north, while dry season use extends toward the south. C2, for its part, consists of dozens of concentrations of intensive land use that are spread over the southern half of ENV. Many of these concentrations are seasonal in their use. Those that scatter in the north central part of C2 attract NOMADs in the rainy season, whereas in the dry season, the main concentrations are found in the southeastern part or the southwestern part of the cluster. The latter parts also contain the sites that provide a fairly stable supply of grazing resources across the seasons. Whether seasonal or not, these ‘key’ resource sites and their exploitation by NOMADs largely condition the spatio-temporal dynamics of the land use in ENV.

**Fig 8:**
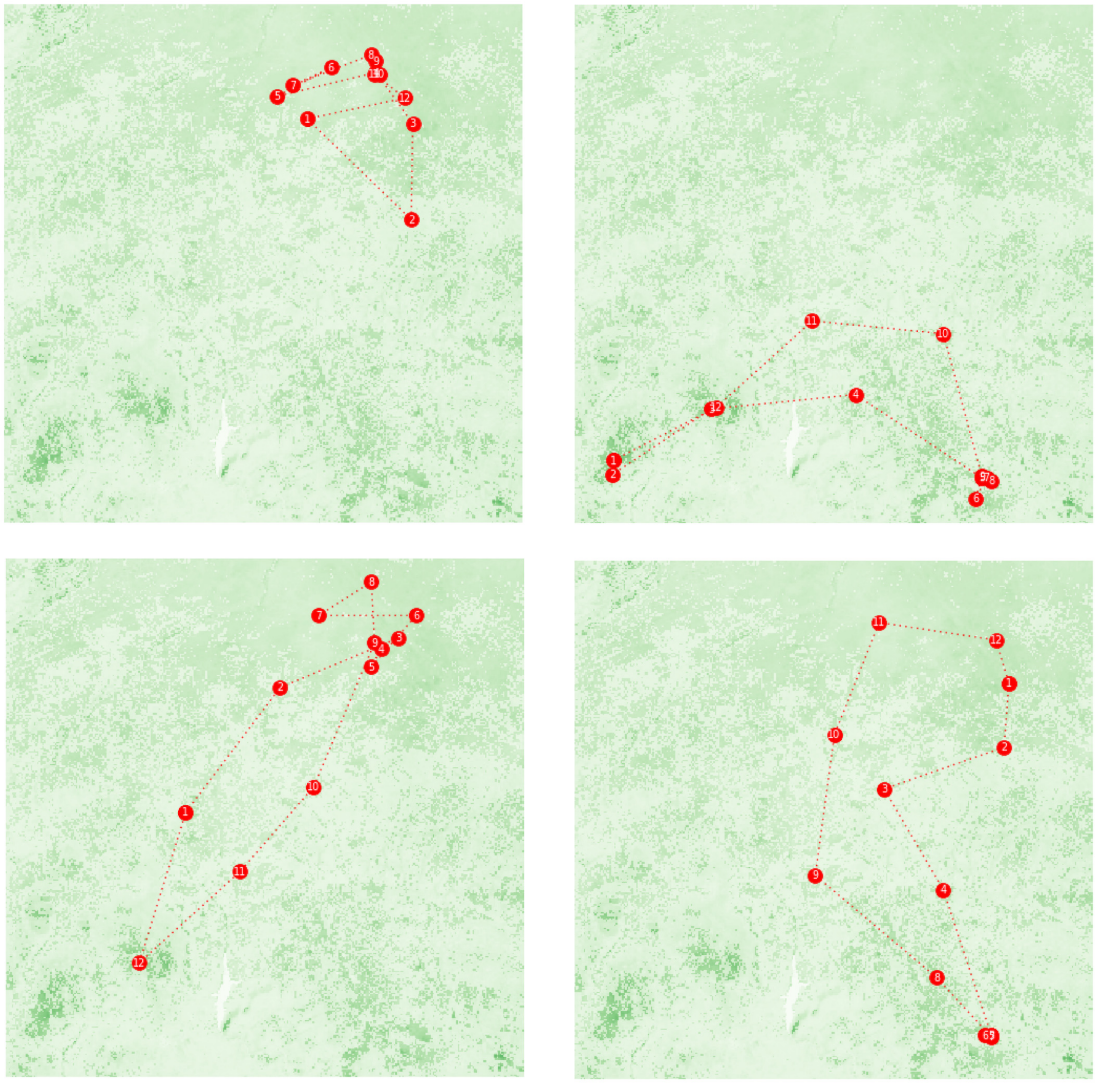
Evolved ROUTEs. Each of the four snapshots was taken at the end of a simulation run; these runs were conducted under identical conditions. NOMAD POPULATION was set to 1 for the purpose of illustration.

At the level of individual agents, NOMADs have evolved distinctive types of ROUTEs in the overall macro pattern of the land use just described. These are depicted in Fig 8, which was derived from another set of simulations. Here, the NOMAD POPULATION was set to be 1 for the purpose of illustration; otherwise, the parameters were identical with the baseline. Each of the snapshots, which were taken after 20,000 years of interactions, plots the successive locations of monthly ‘camping sites’ specified in a NOMAD’s ROUTE, and each circled number denotes the corresponding staying month.

Among the four snapshots in the figure, the two in the top row are the most typical. Each of these ROUTEs exploits the characteristic seasonal variation within either of the land-use clusters: by moving along the north-south gradient of resource availability in C1 in the case of the top left ROUTE and by connecting several major resource sites in C2 in the top right case. The other two ROUTEs in the bottom row, which rely on a much higher level of mobility, cross the boundaries between C1 and C2, taking advantage of the seasonal differences in resource availability found across the clusters. Thus, depending on several parameters such as MOVE RANGE (see below), some degree of freedom was observed in the direction of adaptation that an individual NOMAD could take, even though the broad pattern of land use that emerged at the macro level was mostly stable.

With regard to the resource availability, a NOMAD agent acquired the mean amount of 0.345 units of resources, as measured by adjusted NDVI, per agent per month per run in the 20 runs in the baseline setting. The inter-annual standard deviation from the mean was 0.011 per run, while the standard deviation among the 20 runs was 0.008. These values as well as other similar outputs that appear below were computed using the samples that were obtained in the period from the year 1,001 to 20,000 because of the generally unsettled nature of the model’s behavior in the first few hundred years. The average amount of obtained resources, 0.345, is quite large given what would have been available if the agents could not adapt themselves. For example, an agent with a randomly generated ROUTE that was constrained only by the mobility limit defined by MOVE RANGE (100 km) could access only 0.219 units of resources per month on average. In this additional simulation, each of 20,000 such agents followed its own random ROUTE for one year in turn without any interactions and any adaptation. The standard deviation of the obtained resources among these 20,000 agents was 0.023, which suggests that there is a very large gap in performance between adaptive and not-adaptive agents.

The reported aspects of the model’s behavior, especially those related to pastoral mobility and land use, are largely consistent with what has been observed about actual mobile pastoralists. At the most general level, the model showed the macro pattern of land use that was organized around the spatio-temporal distribution of ‘key’ resource sites in ENV. The importance of these sites for pastoral mobility and livelihoods has repeatedly been emphasized [9,45,80].

The model also captures some of the defining aspects of Fulani pastoralists in West Africa. In many areas in the region, Fulani herders typically stay in the north in the rainy season and then move extensively toward the south as the dry season progresses [20,21,72,73]. This pattern is most closely approximated by the unsettled movement paths along the north-south direction within C1 (e.g., the top-left panel in Fig 8). C1 largely belongs to the arid Sudano-Sahelian ecological zone, which is a traditional niche for Fulani pastoralists. More extensive transhumant routes, such as those documented by Stenning in northern Nigeria, which often stretched over 200 km [21], can also be represented by the comparable ROUTEs that extend across C1 and C2 (e.g., the bottom-left panel in Fig 8).

Moreover, the model also generated the movement trajectories that do not easily fit with the typical north-south transhumance of Fulani pastoralists. Several researchers have noted these discrepancies [20,74]. In this respect, the emergence and persistence of diverse ROUTEs over C2 (e.g., the top-right panel in Fig 8) are especially instructive because they can offer some explanation for the relatively recent tendency of Fulani herders to ‘drift’ to the more humid southern ecological zones [21,73]. To fully understand this aspect of Fulani movement, however, other potentially disrupting factors in these zones should also be accounted for. The most prominent among these factors is the wide presence of tsetse flies and the rapid expansion of cropland, both of which will be discussed below.

### Effects of Parameter Changes

The movement of each individual NOMAD and the resultant resource access to the agent can be affected considerably by changes in the model parameters shown in Table 1. On the other hand, the overall macro pattern of land use that emerges in ENV turns out to be persistent. To detail these trends, the discussion below focuses on four of the parameters: NOMAD POPULATION, CARRYING CAPACITY, MOVE RANGE and GRAZE RANGE. More complete results are given in Supporting Information (S1 Table) and are also briefly discussed later.

**Fig 9:**
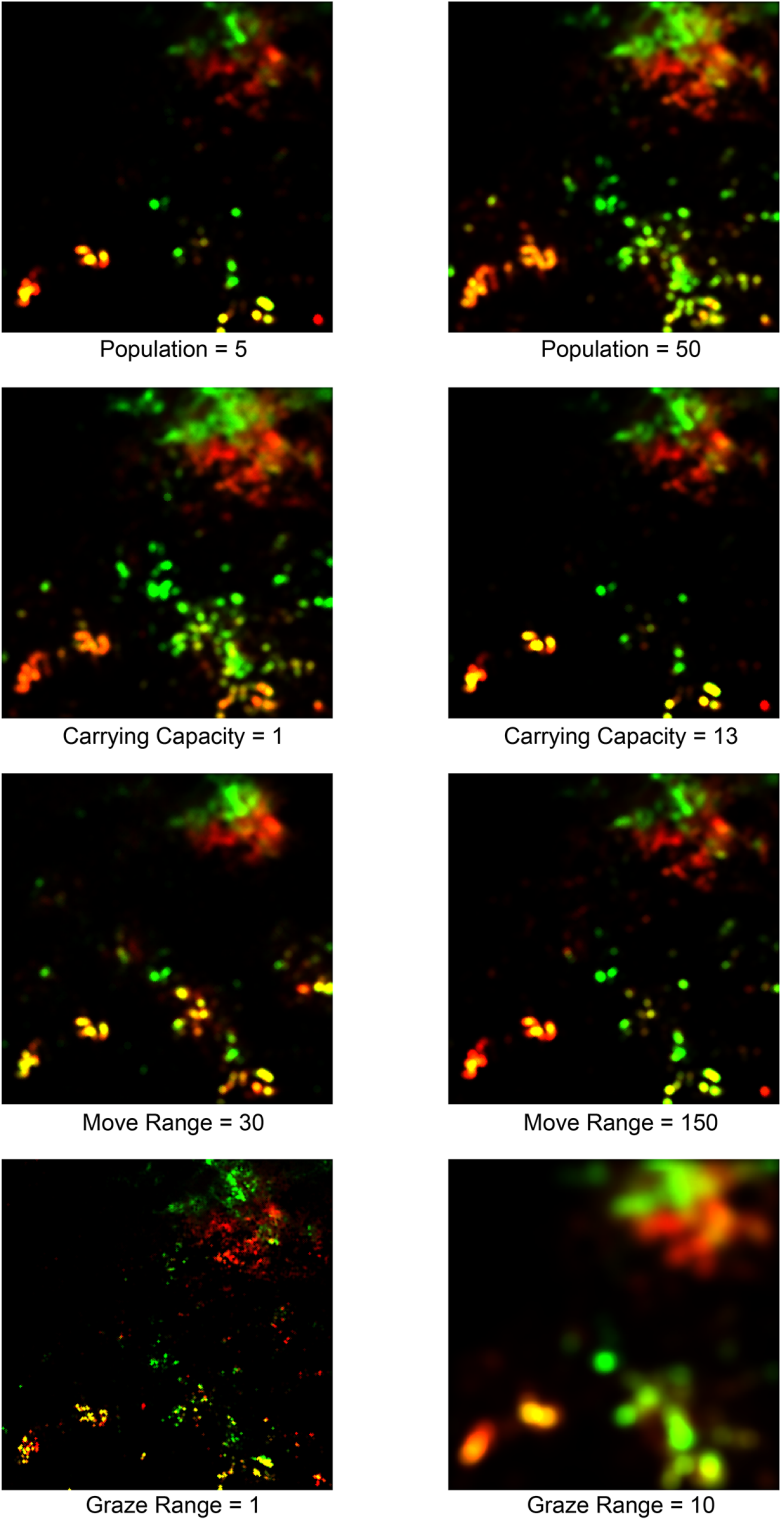
Comparison of distributions of land-use intensity (different parameter conditions). As in Fig 7, each of the maps shown is a composite image of the dry season (January, February and March) and rainy season (July, August and September) distributions of land-use intensity. These maps were derived from simulation runs that were conducted under conditions in which one of the following parameters was changed from the baseline value: NOMAD POPULATION (top row), CARRYING CAPACITY (second row), MOVE RANGE (third row), and GRAZE RANGE (bottom).

The persistency of the land-use pattern can be seen in Fig 9. Each of the composite maps in the figure displays seasonal differences in land-use intensity over ENV obtained under a condition in which one of the parameters is altered from its baseline value. A quick comparison with Fig 7 reveals that the major properties of the land-use pattern in ENV, including the geographical configuration of land-use clusters and seasonal variations in their exploitation, are more or less retained over a wide range of parameter conditions.

**Fig 10:**
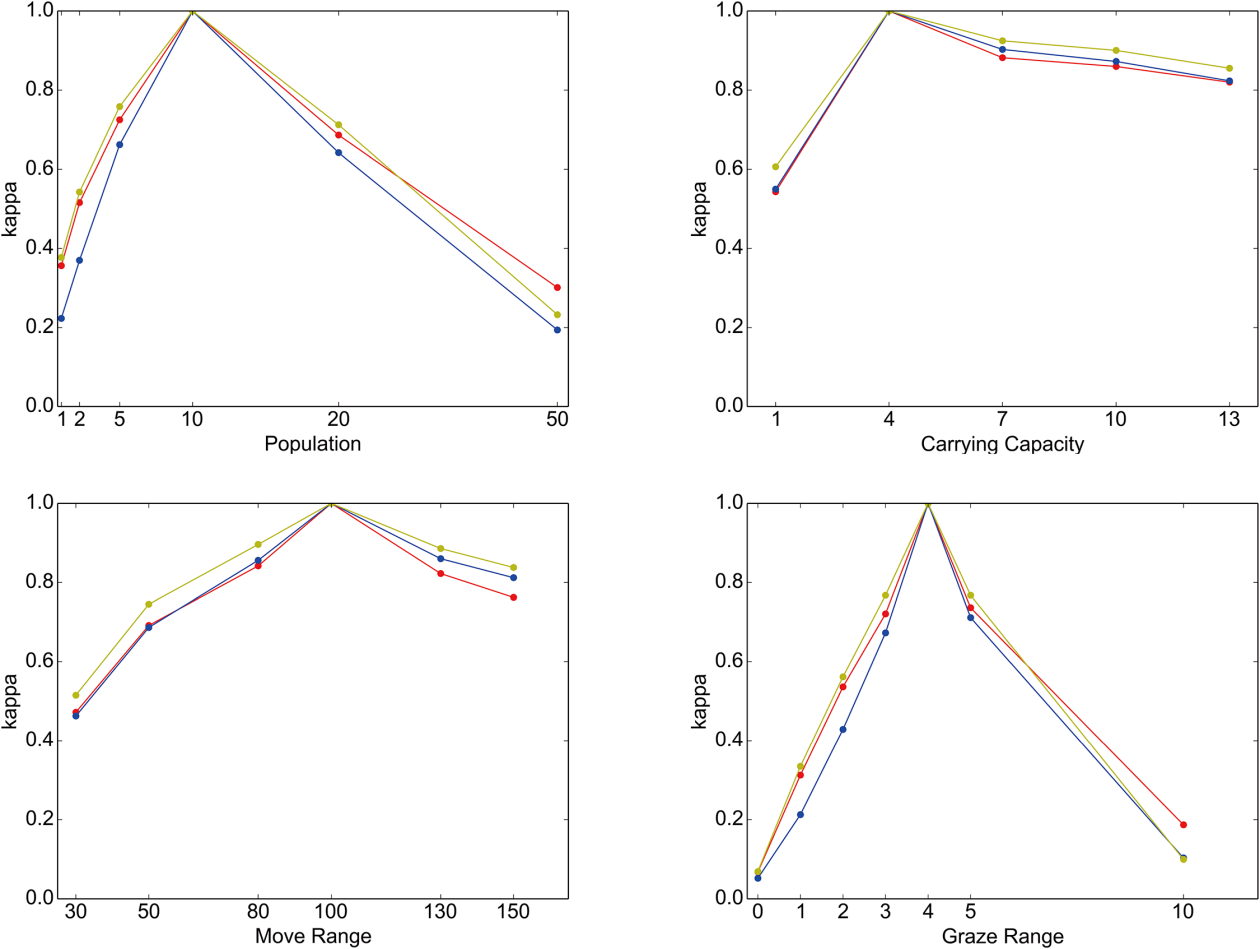
Comparison of kappa statistics. Each of the line graphs shows the dependence of kappa statistics on changes in the following parameters: NOMAD POPULATION (top left), CARRYING CAPACITY (top right), MOVE RANGE (bottom left), and GRAZE RANGE (bottom right). These statistics quantify the similarity of the land-use pattern observed in a given parameter setting to the baseline pattern. These were computed using three different values, 0.001 (red), 0.01 (blue) and 0.1 (yellow), for the land-use threshold.

More rigorous assessment can be done by employing the seasonal land-use categorization introduced above (see Table 2). The idea is to compute a confusion matrix and its kappa statistic (Cohen’s kappa) from two different land-use patterns obtained under two different sets of simulations [81]. Table 3 shows an example of a confusion matrix. This table cross-tabulates the sites in ENV according to how their resources could be seasonally exploited under two different circumstances: the baseline condition (rows, CARRYING CAPACITY = 4) and the case of CARRYING CAPACITY = 13 (columns). The land-use threshold, which separates seasonal from non-seasonal land use, is set to 0.01. A kappa statistic quantifies a degree of similarity between two land-use patterns from the information contained in a confusion matrix. Its value, which is derived from Table 3, is 0.823 (against the maximum of 1.0), which indicates a very high level of agreement between the land-use patterns obtained under these two conditions. Similar calculations lead to the results illustrated in Fig 10, which plots the kappa statistics that are computed against the baseline land-use pattern with different combinations of thresholds and parameters. The figure shows that these statistics are highly robust against the choice of a threshold value for the land-use classification. In contrast, the kappa statistics more or less monotonically deviate from the baseline value (1.0) as each of the model parameters changes. Nonetheless, except for outlying cases, especially in GRAZE RANGE, an observed land-use pattern displays at least moderate similarity (kappa > 0.4) to the baseline pattern. These observations indicate a certain degree of constraints that the ecological patterns and dynamics of ENV impose on the spatial behavior of NOMADs.

**Table 3:** Example of a confusion matrix.

	Dry Season (CC = 13)	Wet Season (CC = 13)	Not Seasonal (CC = 13)	Not Exploited (CC = 13)	Total
Dry Season (CC = 4)	4617	33	613	0	5263
Wet Season (CC = 4)	34	3554	587	16	4191
Not Seasonal (CC = 4)	587	352	12734	4611	18284
Not Exploited (CC = 4)	0	0	628	76610	77238
Total	5238	3939	14562	81237	104976

CC: CARRYING CAPACITY; CC = 4 is the baseline setting. The land-use threshold is set to 0.01.

**Fig 11:**
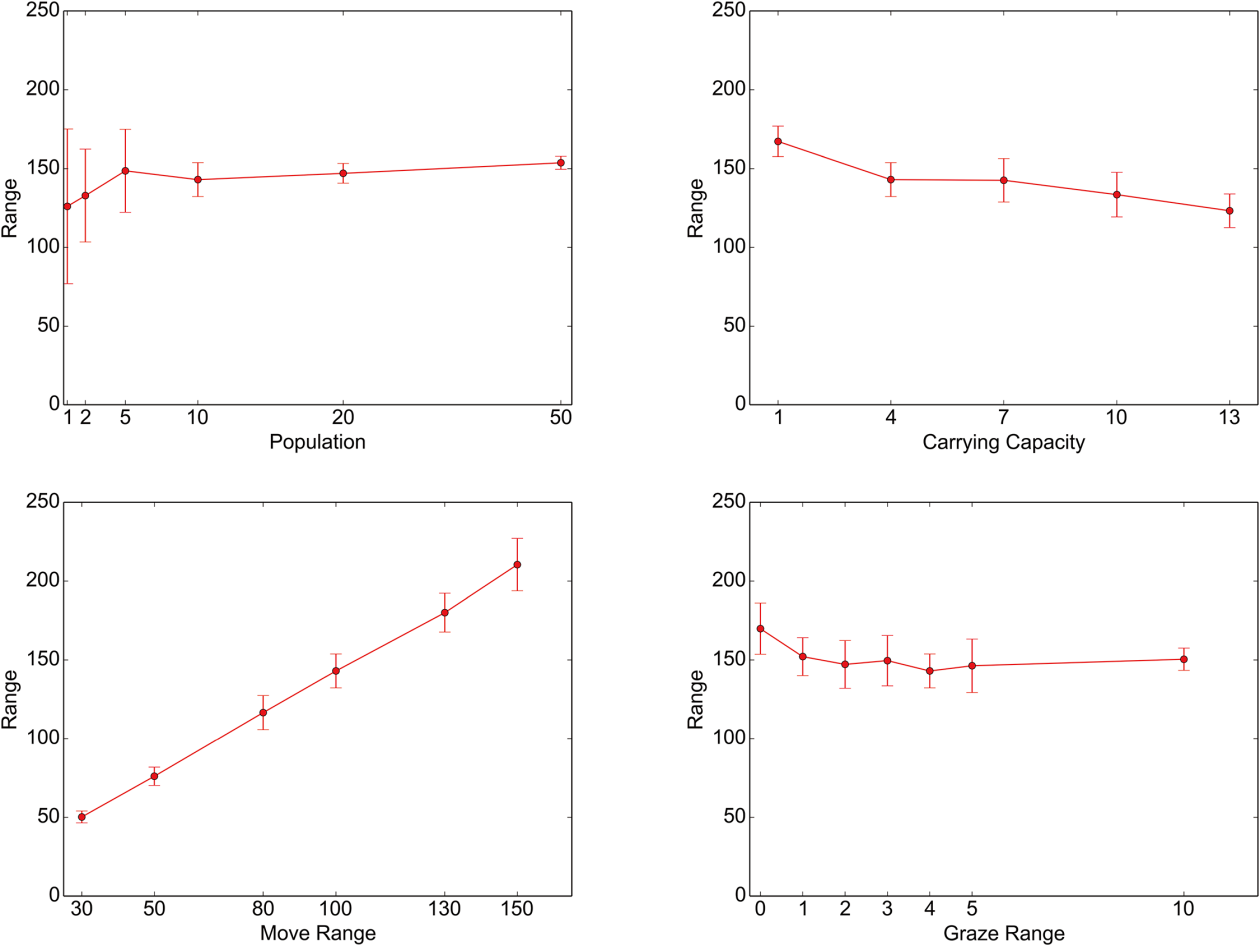
Effects of parameter changes on movement. Each of the line graphs shows the dependence of the mean ROUTE range on changes in the following parameters: NOMAD POPULATION (top left), CARRYING CAPACITY (top right), MOVE RANGE (bottom left), and GRAZE RANGE (bottom right).

**Fig 12:**
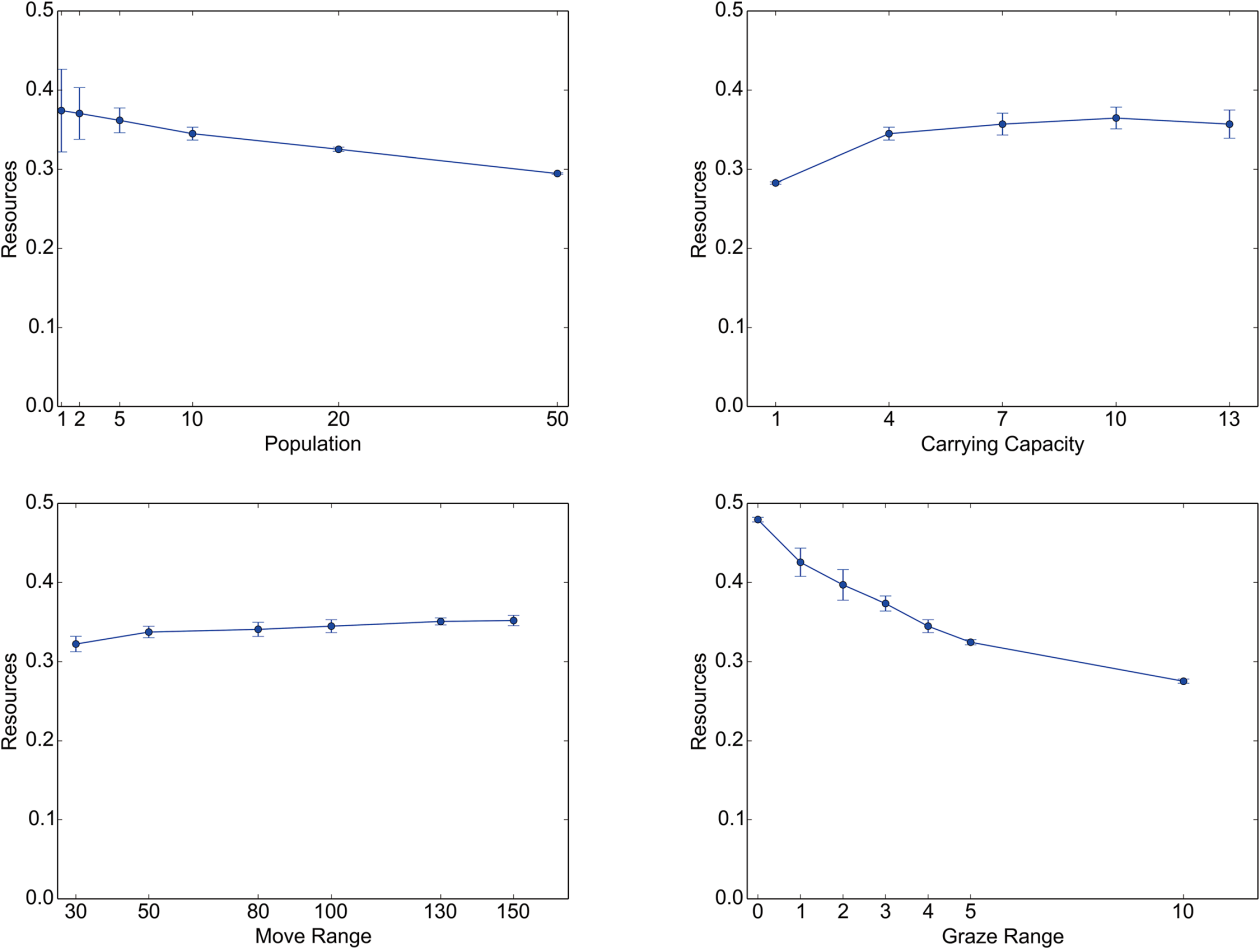
Effects of parameter changes on resource access. Each of the line graphs shows the dependence of the mean obtained resources on changes in the following parameters: NOMAD POPULATION (top left), CARRYING CAPACITY (top right), MOVE RANGE (bottom left), and GRAZE RANGE (bottom right).

Under these constraints, individual agents were affected differently by the change in each of the parameters. Figs 11 and 12 illustrate these effects (see S1 Table for further details). These figures show the dependence of two summary variables, the mean range of their ROUTEs (Fig 11) and the mean amount of resources acquired by the agents (Fig 12), when each of the four parameters examined was changed. The range of a ROUTE is defined as the distance between the two sites along that ROUTE that are the farthest apart from one another. The error bars indicate the standard deviations of these variables among 20 runs.

According to the top left graphs in these figures, increasing NOMAD POPULATION generally enlarges the ranges of ROUTEs (but not necessarily in a statistically significant way; see S1 Table) and monotonically reduces the amount of grazing resources that NOMADs can obtain. Because of the heightened population pressures, especially around the key resource sites, the agents now must search a larger area for grazing resources, which necessitates the exploitation of less endowed sites. This trend is also reflected in the somewhat diffusive land-use pattern shown in the top right map (NOMAD POPULATION = 50) in Fig 9. Similar effects can be obtained by decreasing the CARRYING CAPACITY, which raises the agent population pressure (see the top right graphs in Figs 11 and 12).

The bottom left graph in Fig 11 shows that an increased MOVE RANGE implies an increased range of a ROUTE. This relationship appears to be obvious, but it should be noted that, as the corresponding panels in Figs 9 and 10 show, the resulting land-use pattern at large was not changed much. This observation suggests that there is some redundancy in that an increased level of mobility does not necessarily lead to better resource access. In effect, as the bottom left graph in Fig 12 shows, the mean amount of acquired resource increases as MOVE RANGE increases, but not very markedly.

Finally, raising GRAZE RANGE causes a noticeable decrease in the mean amount of resources available to the NOMADs (see the bottom right graph in Fig 12). This finding is due to the averaging effects of increasing the number of sites included in the calculation rather than any profound change in the behavior of the agents. In fact, as the bottom right graph in Fig 11 tells, the effects of this parameter on NOMAD movement are ambiguous, except for the somewhat extreme case of GRAZE RANGE = 0.0.

Similar analyses can be extended to other aspects of the model. S1 Table displays the main simulation outputs obtained in much broader parametric settings along with their statistical differences from the baseline result. The table also shows the possible effects of alternative specifications of the input data and the behavioral rule, changes that are more extensive than the mere parameter manipulation covered in Table 1. One notable result in this regard is the model’s robustness to the temporal duration of the NDVI data stream. Except for the settings that incorporate just one or two years of vegetation changes, different combinations of NDVI data spanning different periods of time lead the model to highly similar macro-behavior, not only in terms of the emergent land-use pattern (as measured by kappa statistics), but also in relatively sensitive aspects such as the mean amount of obtained resources. This indicates the ‘representative’ nature of the 10-year data stream employed above among possible ecological dynamics that the available MODIS NDVI dataset can generate. Overall, these additional simulations again confirm the ecological constraints of ENV on the seasonal land use of NOMADs. Note also that, in a broad array of settings, the level of resource access that the agents can attain remain much higher than what could be achieved without adaptation, even though specific numbers often deviate significantly from the baseline result.

### Disruptions and Constraints

**Fig 13:**
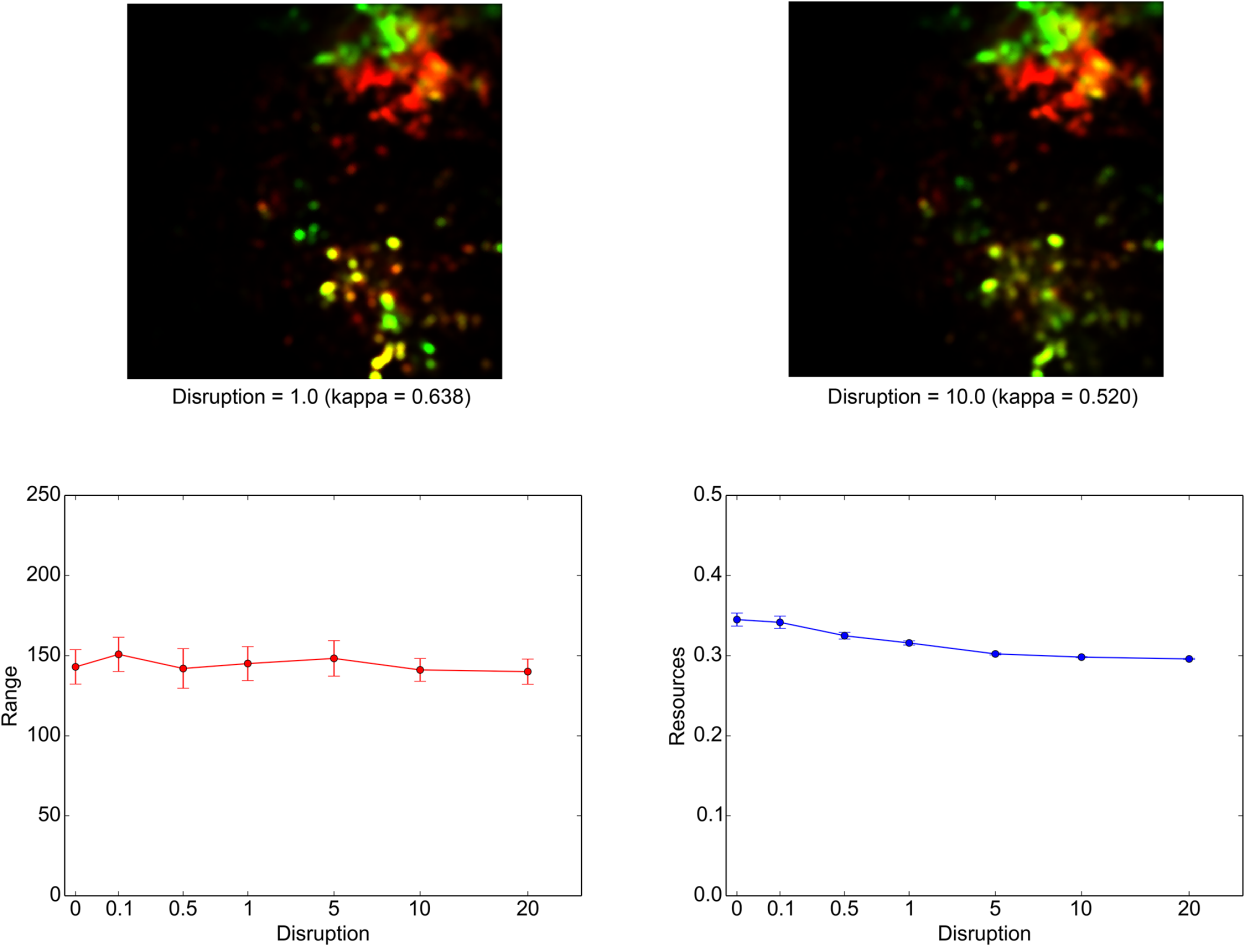
Effects of tsetse flies on the model. The seasonal land-use maps with the corresponding kappa statistics (top row), the means of the ROUTE ranges (bottom left), and the means of the resources available to NOMADs (bottom right) are displayed. The kappa statistics were calculated by setting the land-use threshold to 0.01. These graphs, which are similar to Figs 9 and 11, were derived from simulations that were conducted with different values of DISRUPTION EFFECT; otherwise, the settings were identical to the baseline condition.

Fig 13 illustrates the effects of the tsetse fly disruption on the overall behavior of the model. Here, simulations were run in the baseline condition with the additional effects of tsetse fly presence, which were controlled by DISRUPTION EFFECT. The composite maps of land-use intensity in the top row clearly show that even a relatively small amount of the agent sensitivity to the tsetse presence can cause noticeable alteration in the land-use pattern in ENV, especially in C2 in the south. Although the corresponding kappa statistics still indicate a moderate level of agreement with the baseline land-use pattern at the macro level, the notable feature in this case is the spatially concentrated nature of the discrepancies. Specifically, the hitherto stable sources of grazing resources, which were mainly concentrated in the southwestern part of C2, had now become effectively inaccessible because of the overlap with the distribution of *Morsitans*. The loss of these key sites significantly disturbed the dry season resource access in C2, which even led to a slight increase in the dry season land use in C1. Although the impact of this disturbance on the movement patterns of individual NOMADs is not clear from the bottom left graph in Fig 13, the right graph unambiguously demonstrates its negative effects on the amount of resources that each agent can obtain.

These results make clear how disease vectors such as tsetse flies can worsen pastoral livelihoods over a wide area through the disruption of the underlying land-use pattern in the area concerned. Historically, the wide presence of tsetse flies actually posed a major obstacle to the southern advancement of Fulani herders into more humid ecological zones such as Sudanian and Sudano-Guinean zones. That advancement eventually took place, but only after adaptive measures such as crossbreeding were taken on the part of the herders [20,73]. Fulani pastoralists now widely crossbreed their original zebu cattle with more tsetse-resistant taurin breeds from humid areas. In the context of the model, such a measure can significantly lower DISRUPTION EFFECT and, thus, contribute to an increased level of resource availability through the expansion of accessible sites.

At the same time, some caution is required in interpreting these results because the spatial distribution of the tsetse flies generally shows temporal changes, intra-annually as well as inter-annually. These changes are not considered in the FAO’s dataset used here. Actual pastoralists fully recognize such dynamics and adjust their movements accordingly [75]. Recent efforts to develop more fine-grained spatial data on tsetse distributions [82] can be helpful in incorporating this temporal dimension into the current model.

**Fig 14:**
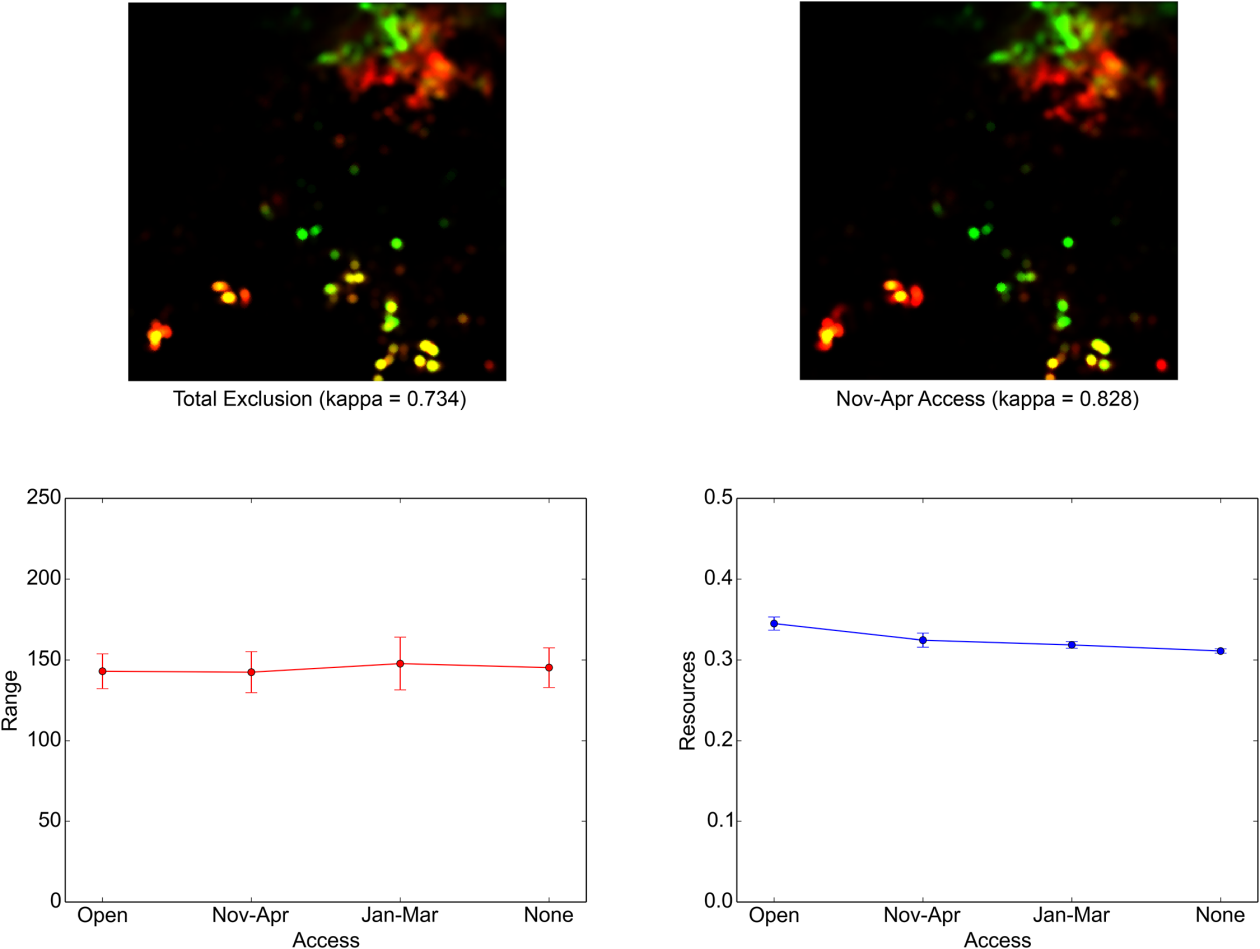
Effects of cropland expansion on the model. The seasonal land-use maps with the corresponding kappa statistics (top row), the means of the ROUTE ranges (bottom left), and the means of the resources available to NOMADs (bottom right) are displayed. These, which are comparable to Fig 13 above, were derived from the simulations conducted at different levels of temporal control of the agricultural land access; otherwise, the settings were identical to the baseline conditions.

Last, the vast expanse of croplands can have more subtle but equally disruptive effects on pastoral resource access. Fig 14 summarizes the simulation results, which were obtained at different levels of temporal control of the access to the agricultural lands in ENV: open access (identical with the baseline condition), half-year access from November to April, dry season access from January to March, and the total exclusion from the croplands. The land-use intensity maps in the top row indicate that tightening or relaxing the land access does not have noticeable effects on the macro pattern of land use in ENV, although some losses of key sites in the south are barely recognizable in the case of the total exclusion. Quantitatively, even in this drastic case, the kappa statistic still remains 0.734, which suggests a substantial level of similarity with the baseline seasonal land use. The effects on the mean range of the ROUTEs are equally ambiguous, as the bottom left graph shows. In contrast, from the bottom right graph, it is clear that tightening the access steadily reduces the mean amount of resources that are available to NOMADs. This trend can be attributed to the near-ubiquitous existence of cropland all over the southern part of the study area, which uniformly reduced the available resources there without markedly altering the spatial pattern of land use.

The expansion of agricultural lands in the drylands and the resulting disruptions in pastoral livelihoods are almost a global phenomenon [18]. The above results quantitatively confirm this aspect of contemporary pastoralism in the context of northeastern Nigeria. Although the NOMADs managed to retain their characteristic land-use pattern, the sheer extent of cropland expansion overwhelmed any effort to obtain grazing resources. In West Africa, negotiated access to croplands, especially in the dry season, has been widely observed [9,73,74]. The simulations suggest that to significantly mitigate the potential loss of available resources, such access must be assured extensively in both its temporal duration and spatial expanse.

## Conclusions

In summary, the above simulations revealed the strong tendency of the model to generate a specific macro pattern of pastoral land use that has a certain configuration of geographical clusters and a certain dynamics of seasonal variations. This pattern, which is reminiscent of the known behavior of Fulani pastoralists in several important respects, ensured a relatively high level of resource access for the NOMAD agents who inhabit the unpredictable landscape. Moreover, the land-use pattern was consistently observed in a wide range of parametric settings, even though the details changed depending on specific parameter values. Finally, the same macro pattern turned out to offer a useful basis for understanding the potential impact of major disrupting factors, such as livestock disease vectors and agricultural land expansion. These factors often altered the land-use pattern in terms of its form and generally hindered resource access for the agents as a result. Nevertheless, the underlying macro dynamics of the model was clearly persistent.

This analysis can be made more extensive as well as more in-depth. The possible combinations of parameters and scenarios are open-ended, and various tools for data analysis can aid the exploration of these possibilities. In particular, the arguments could be made more robust with more systematic sampling of the parameter space than attempted here. Nevertheless, the above analysis has already accomplished much with regard to its major purpose: to illuminate the outstanding strengths of the approach presented here. First, the use of multi-temporal MODIS vegetation data allows a considerable amount of the spatial heterogeneity and temporal variability of actual dryland ecologies to be incorporated into analyses of mobile pastoralism. Second, on this firm basis, the agent-based model enables an extensive investigation of pastoral mobility and resource access, which derives a range of possibilities in which pastoralists can find themselves under different circumstances. Such an investigation might even reveal some robust pattern among these possibilities. For example, there may be strong convergence to a specific configuration of seasonal land use such as the pattern illuminated here. These possibilities and patterns can offer a solid intellectual basis for enhancing the sustainability of mobile pastoralism, which faces numerous risks and threats in the contemporary world. Third, the approach has a high level of applicability and extendibility. The global coverage of MODIS and its public availability make it fairly straightforward to apply the same approach to other dryland areas. Moreover, by combining the MODIS data with other compatible datasets such as those derived from Advanced Very High Resolution Radiometer (AVHRR), which has been in operation for more than 30 years, it is possible to enlarge the time horizon of an analysis still further. Thus, the present approach can be fruitfully applied to an assessment of the possible impacts on pastoral livelihoods of factors working at even larger temporal scales, including the pressing issue of global climate change among other examples.

The enumerated strengths of the present approach are clear advantages over the existing approaches that are found in the literature on pastoralism. Most of the existing approaches, whether qualitative description or quantitative measurement, have not provided adequate spatio-temporal coverage for analyzing a highly variable system such as dryland pastoralism. This circumstance does not mean, however, that meaningful dialogue among the approaches is impossible. On the contrary, there is ample opportunity for mutual enhancement, even the synthesis, of different methodologies. With regard to the model presented here, as was indicated above, the behavioral rule of an agent must be constantly checked against field observations of the corresponding micro-behavior of actual pastoralists. More detailed representation of pastoral livelihoods might become necessary along the way, from more explicit treatment of livestock and human demography to richer description of collaborations and competitions among pastoralists and other relevant actors. Moreover, the recent development of tracking technologies has paved the way for more direct validation of the model. At the very least, quantitative tracking data on pastoralist or livestock movements can be utilized to calibrate some of the parameters of the model, which leads to a potentially significant reduction in the parameter space to be searched in the simulations. Achieving a higher level of efficiency through the use of these data is one fruitful step that can be taken next.

## Supporting Information

S1 Table Summary of sensitivity analysis. The shaded cells correspond to the baseline runs. The mean outputs (ROUTE's range and obtained resources) of 20 runs in each parameter setting were computed along with the standard deviations (SD). Their difference from those in the baseline was assessed by a two-sample Welch’s t-test (*: significant at the 0.05 level; **: significant at the 0.01 level; ***: significant at 0.005 level). The kappa statistic measures the degree of similarity of a seasonal land-use pattern observed in a given parameter setting to the baseline pattern. The underlying confusion matrix was formed using one of three different thresholds (0.001, 0.01 and 0.1); if the mean frequency of visits to a site in the rainy season (the dry season) exceeded that in the dry season (the rainy season) by more than the threshold amount, the concerned site was categorized as 'rainy-season dominant' ('dry-season dominant'). See the main text for more details.
Apart from the parameters and settings that are discussed in the main text, the following conditions were also manipulated: NDVI Years: the temporal range of MODIS NDVI data employed in simulation. NDVI Reduction: the discount rate applied to NDVI values of the land types not preferred by NOMADs (i.e., land types other than ‘grasslands’, ‘savannas’, and ‘woody savannas’). Resource Share: if TRUE, NOMADs which happen to stay on the same site equally share the grazing resources there; if FALSE, the resources are available only to a randomly selected one of them. Movement Cost Function: different ways of computing the cost incurred during a NOMAD's movement, comparing the original step function and alternative linear cost functions with different slope values (per km).
(PDF)
