# Supplementary material for: Computational Research on Mobile Pastoralism Using Agent-Based Modeling and Satellite Imagery
Source: PLoS One. 2016 Mar 10;11(3):e0151157. doi: 10.1371/journal.pone.0151157 (PMC4786315; doi:10.1371/journal.pone.0151157)
Supplement: S1 Table — The shaded cells correspond to the baseline runs. The mean outputs (ROUTE's range and obtained resources) of 20 runs in each parameter setting were computed along with the standard deviations (SD). Their difference from those in the baseline was assessed by a two-sample Welch’s t-test (*: significant at the 0.05 level; **: significant at the 0.01 level; ***: significant at 0.005 level). The kappa statistic measures the degree of similarity of a seasonal land-use pattern observed in a given parameter setting to the baseline pattern. The underlying confusion matrix was formed using one of three different thresholds (0.001, 0.01 and 0.1); if the mean frequency of visits to a site in the rainy season (the dry season) exceeded that in the dry season (the rainy season) by more than the threshold amount, the concerned site was categorized as 'rainy-season dominant' ('dry-season dominant'). See the main text for more details. Apart from the parameters and settings that are discussed in the main text, the following conditions were also manipulated: NDVI Years: the temporal range of MODIS NDVI data employed in simulation. NDVI Reduction: the discount rate applied to NDVI values of the land types not preferred by NOMADs (i.e., land types other than ‘grasslands’, ‘savannas’, and ‘woody savannas’). Resource Share: if TRUE, NOMADs which happen to stay on the same site equally share the grazing resources there; if FALSE, the resources are available only to a randomly selected one of them. Movement Cost Function: different ways of computing the cost incurred during a NOMAD's movement, comparing the original step function and alternative linear cost functions with different slope values (per km). (PDF) [file pone.0151157.s001.pdf]

Population

Value	Range Mean	Range SD	Resources Mean	Resources SD	kappa (thres=0.001)	kappa (thres=0.01)	kappa (thres=0.1)
1	126.04516	49.03017	0.37418*	0.05223	0.35643	0.22342	0.37750
2	132.88539	29.42733	0.37054***	0.03266	0.51590	0.37005	0.54275
5	148.61343	26.29986	0.36176***	0.01565	0.72490	0.66205	0.75848
10	143.01448	10.76324	0.34494	0.00821	1.00000	1.00000	1.00000
20	147.01513	6.25178	0.32526***	0.00274	0.68643	0.64185	0.71237
50	153.69164***	4.13176	0.29471***	0.00090	0.30126	0.19416	0.23269

Carrying Capacity

Value	Range Mean	Range SD	Resources Mean	Resources SD	kappa (thres=0.001)	kappa (thres=0.01)	kappa (thres=0.1)
1	167.20417***	9.69305	0.28277***	0.00149	0.54332	0.54987	0.60644
4	143.01448	10.76324	0.34494	0.00821	1.00000	1.00000	1.00000
7	142.62373	13.77335	0.35700***	0.01380	0.88186	0.90277	0.92467
10	133.50596*	14.18628	0.36469***	0.01371	0.85971	0.87241	0.90038
13	123.26777***	10.66611	0.35702*	0.01773	0.82001	0.82340	0.85531

Move Range

Value	Range Mean	Range SD	Resources Mean	Resources SD	kappa (thres=0.001)	kappa (thres=0.01)	kappa (thres=0.1)
30	50.19804***	3.79795	0.32237***	0.00968	0.47230	0.46271	0.51538
50	76.03863***	5.84265	0.33755***	0.00718	0.69159	0.68655	0.74527
80	116.60178***	10.85238	0.34091	0.00896	0.84196	0.85603	0.89613
100	143.01448	10.76324	0.34494	0.00821	1.00000	1.00000	1.00000
130	180.01595***	12.31534	0.35092**	0.00440	0.82248	0.86009	0.88579
150	210.44363***	16.61023	0.35208***	0.00654	0.76237	0.81201	0.83784

Graze Range

Value	Range Mean	Range SD	Resources Mean	Resources SD	kappa (thres=0.001)	kappa (thres=0.01)	kappa (thres=0.1)
0	169.86273***	16.22744	0.47944***	0.00291	0.06798	0.05192	0.06922
1	152.12900*	12.00073	0.42546***	0.01788	0.31279	0.21295	0.33512
2	147.19625	15.13492	0.39702***	0.01932	0.53632	0.42868	0.56177
3	149.60034	15.97471	0.37353***	0.00954	0.72080	0.67284	0.76763
4	143.01448	10.76324	0.34494	0.00821	1.00000	1.00000	1.00000
5	146.33296	16.96760	0.32466***	0.00330	0.73635	0.71151	0.76772
10	150.46569*	7.09522	0.27539***	0.00272	0.18712	0.10331	0.09971

Alternative Routes

Value	Range Mean	Range SD	Resources Mean	Resources SD	kappa (thres=0.001)	kappa (thres=0.01)	kappa (thres=0.1)
2	146.39215	12.71444	0.32776***	0.00866	0.57824	0.60364	0.63620
10	143.15062	10.44827	0.33500***	0.00836	0.78218	0.80929	0.83369
50	139.91146	14.68052	0.33893*	0.00934	0.87578	0.88979	0.91836
100	143.01448	10.76324	0.34494	0.00821	1.00000	1.00000	1.00000
200	149.49318	12.53761	0.34948	0.00719	0.89864	0.91913	0.93924

Stochastic Noise (Max)

Value	Range Mean	Range SD	Resources Mean	Resources SD	kappa (thres=0.001)	kappa (thres=0.01)	kappa (thres=0.1)
0.001	129.15522***	8.99312	0.23500***	0.01664	0.14321	0.14983	0.22354
0.002	152.14228*	16.45995	0.35500***	0.01023	0.68142	0.69718	0.73506
0.005	146.09454	13.46583	0.35408***	0.00876	0.76538	0.78981	0.81332
0.01	143.01448	10.76324	0.34494	0.00821	1.00000	1.00000	1.00000
0.02	143.39040	13.88132	0.33306***	0.00597	0.67406	0.68211	0.70703
0.05	143.81836	5.39921	0.28215***	0.00309	0.16923	0.17611	0.17566
0.1	148.00524	2.60746	0.24972***	0.00423	0.08399	0.06786	0.07901

Tsetse Disruption

Value	Range Mean	Range SD	Resources Mean	Resources SD	kappa (thres=0.001)	kappa (thres=0.01)	kappa (thres=0.1)
0	143.01448	10.76324	0.34494	0.00821	1.00000	1.00000	1.00000
0.1	150.85777*	10.65081	0.34154	0.00773	0.87561	0.88538	0.91265
0.5	142.05114	12.33936	0.32494***	0.00405	0.70631	0.70386	0.75973
1	145.09663	10.61662	0.31599***	0.00263	0.62287	0.63769	0.67259
5	148.33421	11.12710	0.30231***	0.00122	0.51610	0.53245	0.56218
10	141.16704	7.12922	0.29830***	0.00066	0.50965	0.52042	0.55153
20	140.06948	7.86011	0.29603***	0.00068	0.49687	0.51070	0.53646

Cropland Access

Value	Range Mean	Range SD	Resources Mean	Resources SD	kappa (thres=0.001)	kappa (thres=0.01)	kappa (thres=0.1)
Open	143.01448	10.76324	0.34494	0.00821	1.00000	1.00000	1.00000
November to April	142.47123	12.73135	0.32446***	0.00864	0.81607	0.82784	0.87527
January to March	147.76633	16.29439	0.31867***	0.00416	0.79710	0.81569	0.85707
None	145.28108	12.28822	0.31119***	0.00272	0.72277	0.73369	0.79766

NDVI Years

Value	Range Mean	Range SD	Resources Mean	Resources SD	kappa (thres=0.001)	kappa (thres=0.01)	kappa (thres=0.1)
2003-2014	146.97169	13.80336	0.34719	0.00793	0.88846	0.92033	0.95217
2004-2014	139.61138	9.94264	0.34300	0.00826	0.89357	0.90704	0.93922
2005-2014	143.01448	10.76324	0.34494	0.00821	1.00000	1.00000	1.00000
2006-2014	141.31025	9.89819	0.34696	0.00525	0.89813	0.91781	0.94758
2007-2014	139.13013	11.50173	0.34474	0.00580	0.86968	0.88479	0.92667
2008-2014	150.87517	16.25242	0.34629	0.00495	0.85356	0.87654	0.91743
2009-2014	146.91090	17.77292	0.34375	0.00712	0.85434	0.87185	0.91837
2010-2014	142.90099	8.82769	0.34377	0.00532	0.83916	0.85290	0.90951
2011-2014	146.15097	18.51903	0.34277	0.00873	0.81875	0.83561	0.90488
2012-2014	139.79105	13.84852	0.34588	0.00528	0.78209	0.80925	0.89073
2013-2014	152.17096	18.86282	0.34990*	0.00398	0.75233	0.78469	0.86187
2014	144.28642	14.24276	0.35827***	0.00701	0.71891	0.72143	0.82368

NDVI Reduction

Value	Range Mean	Range SD	Resources Mean	Resources SD	kappa (thres=0.001)	kappa (thres=0.01)	kappa (thres=0.1)
0	169.73563***	10.86017	0.45020***	0.00159	0.15778	0.15331	0.15406
0.25	165.80583***	8.81847	0.38841***	0.00658	0.61821	0.59624	0.69413
0.5	143.01448	10.76324	0.34494	0.00821	1.00000	1.00000	1.00000
0.75	142.79479	15.66451	0.32263***	0.00757	0.78676	0.79562	0.83667
1	138.40764	15.62632	0.30783***	0.00373	0.70127	0.70604	0.77082

Alternative Specification: Resource Share

Value	Range Mean	Range SD	Resources Mean	Resources SD	kappa (thres=0.001)	kappa (thres=0.01)	kappa (thres=0.1)
TRUE	143.01448	10.76324	0.34494	0.00821	1.00000	1.00000	1.00000
FALSE	147.14684	12.95162	0.34533	0.00858	0.89472	0.92766	0.94917

Alternative Specification: Movement Cost Function

Value	Range Mean	Range SD	Resources Mean	Resources SD	kappa (thres=0.001)	kappa (thres=0.01)	kappa (thres=0.1)
Step Function	143.01448	10.76324	0.34494	0.00821	1.00000	1.00000	1.00000
Linear: slope=0.01	286.04156***	17.96989	0.36256***	0.00369	0.76219	0.81838	0.87137
Linear: slope=0.05	159.61406*	30.52544	0.34684	0.00681	0.79348	0.80362	0.83829
Linear: slope=0.1	94.12274***	13.89143	0.34027*	0.00470	0.72213	0.72118	0.76543
Linear: slope=0.5	28.60233***	1.42803	0.32219***	0.00746	0.43340	0.42472	0.51148
Linear: slope=1.0	20.51898***	0.80697	0.29267***	0.01108	0.33496	0.35124	0.44535

The shaded cells correspond to the baseline runs. The mean outputs (ROUTE's range and obtained resources) of 20 runs in each parameter setting were computed along with the standard deviations (SD). Their difference from those in the baseline was assessed by 2-sample Welch's t-test (*: significant at the 0.05 level; **: significant at the 0.01 level; ***: significant at 0.005 level).
The kappa statistic measures the degree of deviation of a sesonal land-use pattern observed in a given parameter setting from the baseline pattern. The underlying confusion matrix was formed using one of three different thresholds (0.001, 0.01 and 0.1); if the mean freuqency of visit to a site in the rainy season (the dry season) exceeds that in the dry season (the rainy season) by more than the threshold amount, the concerned site was categorized as 'rainy-season dominant' ('dry-season dominant'). See the main text for more details.
Apart from the parameters and settings that are discussed in the main text, the following conditions were also manipulated:
NDVI Years: the temporal range of MODIS NDVI data employed in simulation
NDVI Reduction: the discount rate applied to NDVI values of the land types not preferred by NOMADs (i.e., land types other than 'grasslands', 'savannas', and 'woody savannas')
Resource Share: if TRUE, NOMADs which happen to stay on the same site equally share the grazing resources there; if FALSE, the resources are available only to a randomly selected one of them
Movement Cost Function: different ways of computing the cost incurred during a NOMAD's movement, comparing the original step function and alternative linear cost functions with different slope values (per km)
